# Molecular and functional evolution of the fungal diterpene synthase genes

**DOI:** 10.1186/s12866-015-0564-8

**Published:** 2015-10-19

**Authors:** Marc JC Fischer, Camille Rustenhloz, Véronique Leh-Louis, Guy Perrière

**Affiliations:** Université de Strasbourg, INRA, Inst Natl Recherche Agron, Métab Second Vigne, Unit Mixte Recherche Santé Vigne & Qual Vins, 28 rue de Herrlisheim, F-68021 Colmar, France; Université de Strasbourg, CNRS, FRE 2326, Institut de Biologie Moléculaire et Cellulaire du CNRS, UPR 9002, 15 rue René Descartes, F-67084 Strasbourg, France; Universite Claude Bernard – Lyon 1, 43 bd. du 11 Novembre 1918, Laboratoire de Biometrie et Biologie Evolutive, UMR CNRS 5558, F-69622 Villeurbanne, France

**Keywords:** Diterpene synthase, Horizontal gene transfer, Fungi, Geranylgeranyl diphosphate synthase

## Abstract

**Background:**

Terpenes represent one of the largest and most diversified families of natural compounds and are used in numerous industrial applications. Terpene synthase (TPS) genes originated in bacteria as diterpene synthase (di-TPS) genes. They are also found in plant and fungal genomes. The recent availability of a large number of fungal genomes represents an opportunity to investigate how genes involved in diterpene synthesis were acquired by fungi, and to assess the consequences of this process on the fungal metabolism.

**Results:**

In order to investigate the origin of fungal di-TPS, we implemented a search for potential fungal di-TPS genes and identified their presence in several unrelated Ascomycota and Basidiomycota species. The fungal di-TPS phylogenetic tree is function-related but is not associated with the phylogeny based on housekeeping genes. The lack of agreement between fungal and di-TPS-based phylogenies suggests the presence of Horizontal Gene Transfer (HGTs) events. Further evidence for HGT was provided by conservation of synteny of di-TPS and neighbouring genes in distantly related fungi.

**Conclusions:**

The results obtained here suggest that fungal di-TPSs originated from an ancient HGT event of a single di-TPS gene from a plant to a fungus in Ascomycota. In fungi, these di-TPSs allowed for the formation of clusters consisting in di-TPS, GGPPS and P450 genes to create functional clusters that were transferred between fungal species, producing diterpenes acting as hormones or toxins, thus affecting fungal development and pathogenicity.

**Electronic supplementary material:**

The online version of this article (doi:10.1186/s12866-015-0564-8) contains supplementary material, which is available to authorized users.

## Background

Horizontal Gene Transfer (HGT) is an exchange of genetic material between different strains or species ultimately integrated into the genome. The existence of HGTs has been perceived by the general public as a major health issue, with the recent appearance and spread of bacteria that are multiresistant to antibiotics. The rapid increase in available genomic sequences, combined with the development of analytical techniques, has enabled the detection of numerous events of recent or ancient HGTs between prokaryotes and eukaryotes, as well as within each group of organisms [1]. Following a recent review of the presence of HGT in fungi, it was found that these events are relatively common [2, 3]. The majority of these transfer events involve bacterial [4–6] or fungal donors [7–9]. The transfer of genes between plants and fungi is also possible [10–12], and the transfer of a nitrate assimilation cluster from the *Oomycota* to an ancestral *Dikarya* species was also recently documented [13].

Plants, fungi and bacteria are able to synthesize diterpenes. Sequence analyses, suggest that the di-TPSs from those organisms share a common evolutionary origin resulting from an ancient HGT [14, 15]. Two types of bacterial di-TPSs have been described. Type I di-TPSs initiate a cyclization reaction via heterolytic cleavage of the polyprenyl diphosphate, whereas type II di-TPSs initiate this reaction via protonation of a double bond or an epoxide ring. It has been suggested that both di-TPSs were horizontally transferred from soil bacteria to plants and that an early fusion of bacterial type I and II enzymes led to an increase in size of the encoded protein, from ~500 in bacteria to ~900 aa in plants [16, 17]. However, it cannot not be completely ruled out that a yet undescribed bacterium, already endowed with a fused type I and II di-TPS, may have existed and may have been responsible for this HGT event. TPS genes probably played an important role in the development of land plants, as they made it possible for the gibberellin phytohormones to stimulate growth and trigger developmental processes such as seed germination, flowering, fruit formation and plant senescence [18–20]. In addition, these genes also played a role in the synthesis of molecules involved in plant defence [21]. The gibberellin phytohormone pathway was presumably not fully present in early plants. The moss *Physcomitrella patens* does not produce gibberellins. Instead, it utilizes the diterpene metabolite *ent-*kaurenoic acid, a gibberellin precursor, as its endogenous developmental regulator [19]. The lycophyte *Selaginella moellondorffii* also does not produce gibberellins. However, it encodes the gibberellin signaling pathway in its genome and uses it for spore and sexual organ development [18].

Plant diterpenoids form a very large family, with more than 3,000 distinct structures described thus far [22]. Furthermore, diterpenes belong to the very large isoprenoid family, which includes more than 40,000 described molecules [23]. Isoprenoids are classified by the number of carbons in the skeletal structure: hemi (C5), mono- (C10), sesqui- (C15), di- (C20) and tri- (C30) terpenoids are the most basic, and latex (C > 1,000) the largest representatives. Angiosperms usually have several genes encoding mono-, sesqui- and di-terpenoid synthases. The genome of the embryophyte *P. patens*, the most basal land plant species for which a genome sequence is available, together with data gathered on gymnosperms and angiosperms, have suggested part of the evolutionary history of this family of enzymes [22, 24–26]. Current evidence suggests that early land plants had a single di-TPS gene but no monoterpene or sesquiterpene synthase genes. The ancestral di-TPS gene likely underwent multiple duplication events, as revealed by the presence of only one di-TPS gene in the *P. patens* genome (a bifunctional di-TPS copalyl diphosphate synthase/kaurene synthase, [25]), as opposed to the presence of 66 TPS-like genes in the genome of the later diverging *S. moellondorffii* [27]. Most di-TPSs are approximately 210 aa longer than mono- or sesqui-TPSs, and plant mono- and sesqui-TPSs probably evolved from di-TPSs via the loss of an internal sequence [16]. Most mono- and di- terpenoid synthases also contain an N-terminal plastid transit peptide, whereas this sequence is usually absent from sesqui-TPSs.

It is also noteworthy that gibberellins are not limited to the plant and bacterial kingdoms. Gibberellin was first discovered in the fungus *Fusarium fujikuroi* (=*Gibberella fujikuroi*), and was shown to play an important role in the symptomology of bakanae, an *F. fujikuroi*-incited disease of rice*.* Gibberellins are highly diverse, with 136 analogs described from plants, fungi and bacteria (http://www.plant-hormones.info/gibberellin_nomenclature.htm). Fungal di-TPS enzymes are highly similar in size to plant enzymes, and the combination of biochemistry studies with molecular genetic analyses has facilitated the comparison of plant and fungal biochemical pathways leading to the formation of gibberellins in plants and fungi. The first step in gibberellin biosynthesis in both plants and fungi is catalyzed by a di-TPS, whereas later steps in the biosynthesis differ (post *ent*-7-hydroxy kaurenoic acid). The data indicate that the plant and fungal di-TPSs catalysing the initial steps in gibberellin biosynthesis are similar and therefore share a common evolutionary origin, whereas genes encoding enzymes for the late stage in the pathway most likely have different evolutionary origins in fungi and plants and were not obtained by HGT from plants or bacteria [28, 29]. The recent availability of fungal genomes in large numbers has provided us with the opportunity to investigate the multiple transfers between fungi of genes involved in diterpene synthesis and to discuss the consequences of transfers on fungal metabolism. In the present study, we sought to determine whether these transfers involved clusters of genes acting in the same functional pathway or whether only the di-TPS was transferred. We also found putative di-TPSs in Basidiomycota and sought to investigate the mechanism allowing these fungi to acquire their di-TPS.

## Results

### Distribution of di-TPS genes in fungal genomes

By querying various genome databases covering ca. 1000 fungal species and following a manual curation step of the annotations, we found 57 putative di-TPS sequences. In most cases, the gene annotation predicted/proposed by the fungal genome project was used. Otherwise, the annotation was completed by searching for exon and intron positions. BLAST searches across fungal species generated eight positive hits for putative di-TPSs that were not shortlisted for further analyses, the inferred gene being either too small (*Aulographum hederae* 408580 and 3987706, *Aspergillus kawachii* KAW_01797, *A. niger* XP_00139461, *Neofusicoccum parvum* EOD52872, *Phaeosphaeria nodorum* XP_001792462, *Punctularia strigosozonata* (12-scaffold join 470791–470964; 471015–472545; 472609–472664; 472694–472709), *Rhytidhysteron rufulum* (NODE_501 join 2571–1717; 1618–677) or too large (*Fusarium oxysporum* EGU79219) to correspond to a classical di-TPS (ca 950aa). It is also noteworthy that the sesquiterpenoid trichothecene synthase-like genes, found in several unrelated fungi [30], have weak similarities with di-TPS, especially in their 5’ section. These sequences are very short (around 300aa) and were therefore not considered in our analyses. The list of the 57 fungal di-TPS genes examined in this study is provided in Additional file 1.

**Fig. 1:**
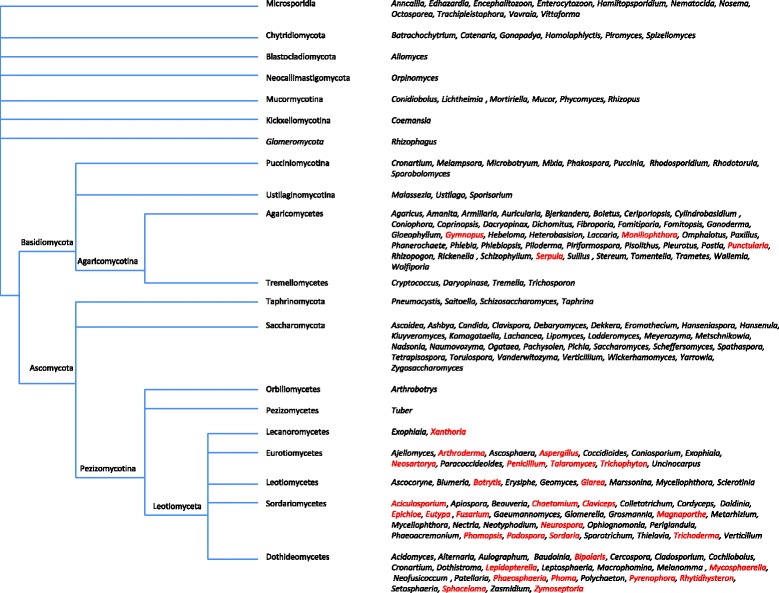
Fungal phylogenetic tree showing genera with genome information. Genera with putative di-TPS genes are indicated in red. For every di-TPS gene accession, the systematic classification found on the genome web page was used for the fungal phylogenetic tree

The 48 fungal species containing di-TPSs genes are not evenly distributed over the fungal phylogeny. In paerticular, none of the species in early diverging lineages of fungi contain a di-TPS (Fig. 1). Di-TPS genes are found exclusively in the *Dikarya* subkingdom (Ascomycota and Basidiomycota)é Furthermore, they are more common across Ascomycota than Basidiomycota: 30 of the 112 Ascomycota genera examined include at least one species with a di-TPS gene in its genome. In contrast, only four of 59 genera of Basidiomycota examined had di-TPS genes. Ascomycota species having di-TPS genes are not uniformly distributed in the Ascomycota tree, since none of the 31 Saccharomycetes genera gave a positive hit for di-TPS. The large number of Sacharomycetes genomes available means that it is unlikely that di-TPS genes are widespread in these organisms. It is not yet known whether di-TPSs are absent from the Pezizomycetes and Orbilomycetes species, because genome sequences for only two genera are available. In view of the small number of genomes available for Pezizomycetes and Orbilomycetes, it is possible that species belonging to these groups may also contain di-TPS genes, which would imply the presence of these genes in all groups from Pezizomycotina. In the case of Basidiomycota, we found only five species (from four genera out of 60) having di-TPS genes, all of which belong to the Agaricomycotina subphylum, comprising four different families and genera. We were able to obtain full-length sequences for *Punctularia strigosozonata* (Corticiales), *Serpula lacrymans* (Boletales) and *Gymnopus luxurians* (Agaricales)*.* In the case of *Moniliophthora perniciosa* and *M. roreri* (Agaricales), the sequences were not included in our analyses since these genome sequence databases (1.9X and 15.0X respectively) have contigs that are too small to contain full-length di-TPS sequences. The *Moniliophthora* di-TPSs are nevertheless expected to be functional, as these fungi are known to produce plant-like hormones, including gibberellins and auxins [31]. According to current knowledge, Tremellomycetes, Ustilaginomycotina and Pucciniomycotina contain only a small number of species for which genomic information is available, and it is thus possible that some species belonging to this group also contain di-TPS genes, which would mean that they are present in Basidiomycota.

**Fig. 2:**
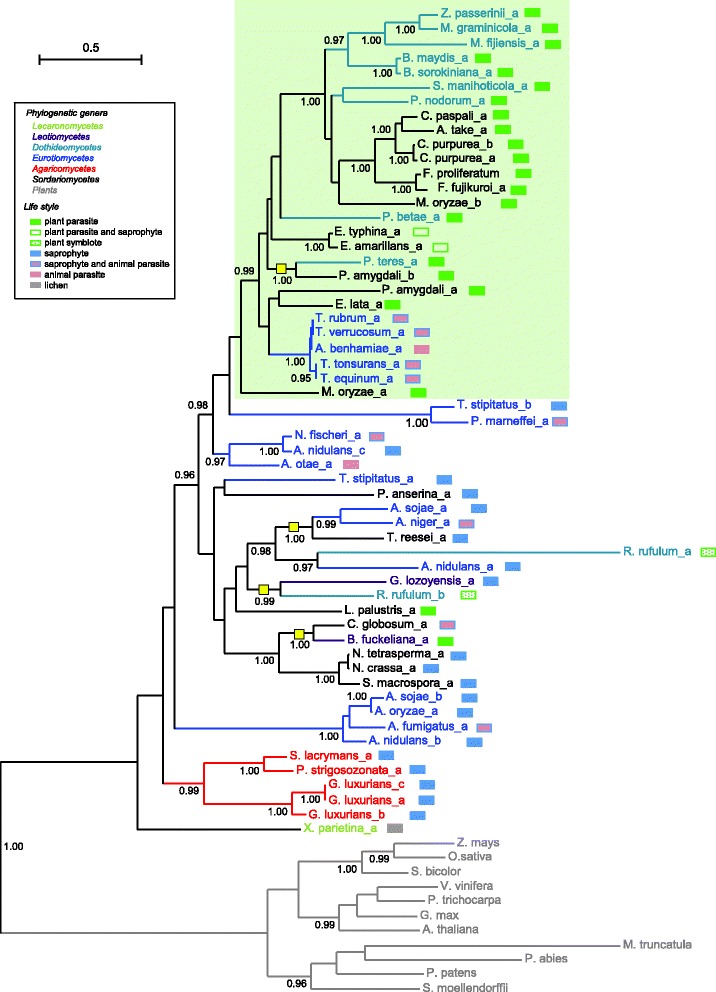
Phylogenetic tree generated from inferred amino acid sequences of fungal and plant di-TPSs. The fungal and plant di-TPS used are listed in Additional file 1. For species possessing multiple di-TPS genes, “_a”,” _b” and “_c” are added to the species designation. The colours used for the species refer to their phylogenetic positions and the oblongs refer to their life style, as described in the legend. The tree is rooted at the level of the plant clade and branch values correspond to aLRT results (only values ≥ 0.95 are shown). The proposed HGT cases are highlighted by yellow squares

The phylogenetic analysis of the 57 selected di-TPS genes (Fig. 2) shows a monophyletic clade of all di-TPS sequences, suggesting that they may have diverged from a common ancestral sequence. Interestingly, some aspects of the topology of the resulting gene clade di-TPS tree are not correlated with the species phylogeny (Fig. 1). The di-TPS phylogeny may to a certain extent be related to the lifestyle of the species. A large group of 27 sequences concentrates 19 of the 22 fungi living in close relationship with plants, among which 17 are plant parasites (Fig. 2, green box). This group does not contain di-TPS sequences from saprophytic fungi. The 11 sequences from animal pathogenic fungi are classed into two groups, one of which is scattered across saprophytic species (six sequences). The other group forms a unique clade in the plant parasite and plant saprophyte group. This clade of five sequences includes the eurotiomycetous species *T. equinum*, *T. verrucosum, T. rubrum, T. tonsurans* and *A. benhamiae* sequences (Fig. 2, pink box) species belonging to *Arthrodermataceae*, suggesting a common ancestral origin (a plant parasitic donor) of di-TPS genes for these species*.*

### Catalytic sites, copy number variation, exon-intron patterns, and functional clusters

Di-TPSs were checked for potential functional catalytic sites. Recent phylogenies from plant TPSs concentrated at the catalytic sites have proposed three major classes of plant di-TPSs (Type I, II, I/II). which can be distinguished by the presence of a certain number of typical features [32]. Type I di-TPSs contain a DDXXD motif and NSE/DTE signature, type II di-TPSs contain a DXDD motif, and bifunctional type I/II di-TPSs contain both DXDD and DDXXD motifs and a NSE/DTE signature. Such bifunctional type I/II di-TPSs are only found in primitive plants, such as *S. moellondorffii* (h subfamily) as well as in some gymnosperms such as *Abies grandis*(d subfamily) [33]. A multiple sequence alignment (Table 1) was performed on the fungal sequences for the Mg^2+^-PPi binding, H^+^-initiated cyclization and Mg^2+^-ionization/cyclization sites already described in plants [16]. Typical bifunctional type I/II signatures were usually found in fungal di-TPS, which is in agreement with biochemical data that found them to be cytosolic bifunctional enzymes [34]. Mg^2+^-PPi binding regions contain the expected D/E residues, except for A. oryzae_a (no D/E residue), C. purpurea_a and B. fuckeliana_a (no alignment in this region). DXDD H^+^-initiated cyclization sites failed to be conserved in only 3 out of 47 cases (P. anserina_a, R. rufulum_a, Z. passerinii_a). In fungi, the Mg^2+^-ionization/cyclization DDXXD site tends to be replaced by a DEXXE site. This DEXXE site was not found in 11 cases (A. nidulans_a, G. lozoyensis_a, L. palustris_a, M. oryzae_b, P. marneffei_a, R. rufulum_a*,* R. rufulum_b*,* T. stipitatus*_*a*,* T. stipitatus_b*,* X. parietina_a*,* Z. passerinii_a). The results found on fungal di-TPSs show that in most cases, all three Mg^2+^-PPi binding, H^+^-initiated cyclization and Mg^2+^-ionization/cyclization sites are conserved, whereas plant di-TPSs having all three sites are uncommon [16].

**Table 1:** Partial alignment of fungal di-TPS highlighting catalytic domains

Fungus Sequence name	Mg^2+^-PPi binding (D/E enriched region after an alpha helix)	H^+^-initiated cyclization (DXDD)	Mg^2+^-ionization/cyclization DEXXE
A. take_a	RDQLGQW*HDVEKSN*HIGVEL	APHTA*DVDDTAK*ALLAL	MLSMFGYQC*DEFIE*TVAGPA
C. paspali_a	QQQLSEW*NDVEITN*HIGVEL	APHTA*DVDDTAK*ALLAL	MLSLLGYQT*DEFIE*TVAGSA
F. fujikuroi_a	KRQLAVW*NDVEDTN*HIGVEF	APRTA*DVDDTAK*ALLAL	YLSLLGYQT*DEYME*AVAGPV
F. proliferatum_a	KRQLAVW*KDVEETN*HIGVEL	APRTA*DVDDTAK*ALLAL	YLSLLGYQT*DEYME*AVAGPV
C. purpurea_b	QLQLSQW*DDVEKSN*HIGVEL	APHTA*DVDDTAK*ALLAL	MLSLLGYQT*DEFIE*AVAGPA
M. oryzae_b	RTQLQNW*DDVLSTN*HIGIEI	APRAP*DADDTAK*GLLAL	LVSMLSYQA*DEFIH*KSAAPA
P. nodorum_a	EEQLIAW*DDVLDTN*HIGVEM	APRAV*DVDDTAK*GLLTL	IISMLGYQI*DEFFE*AEAAPA
S. manihoticola_a	VSQLQQW*DDLVESN*HIGVEL	APRTA*DVDDTAK*GLLAL	LLSMYGYQN*DEFFE*THAMAG
M. graminicola_a	QSQLATW*TDMEATN*HIGVEL	APGTV*DVDDTAK*GLLAM	FMTLINIQV*DEFIE*GIATQA
B. sorokiniana_a	DRQLNAW*NDLVSTN*HIGVEL	VQRAV*DVDDTAK*GLLAL	VMSLLGFQT*DEFFE*AFATPA
B. maydis_a	DRQLNAW*NDLVSTN*HIGVEL	IQRAV*DVDDTAK*GLLAL	VMSLIGFQT*DEFFE*AFATPA
A. benhamiae_a	RSQLEEW*RVSETMH*VGFEII	PDFQS*DMDDTAR*GMIAL	IISFLNYQA*DEFLE*AVAGPH
T. verrucosum_a	RSQLEEW*RVSETMH*VGFEII	PDFQS*DMDDTAR*GMIAL	IISFLNYQA*DEFLE*AVAGPH
T. equinum_a	RSQLEEW*RVSETMV*GFEIIV	PDFQP*DMDDTAR*GMIAL	VISFLNYQA*DEFLE*AVAGPQ
T. rubrum_a	RSQLEEW*RVSETMH*VGFEII	PDFQS*DMDDTAR*GMIAL	IISFLNYQA*DEFLE*AVAGPH
T. tonsurans_a	RSQLEEW*RVSETMH*VGFEII	PDFQP*DMDDTAR*GMIAL	VISFLNYQA*DEFLE*AVAGPQ
P. amygdali_b	RAQLAAL*DVSTTEH*VGFEII	PGFQA*DVDDTAK*TISTL	FIAMLNFQL*DEFME*ATAGIL
E. lata_a	QRQLQNW*DVESSTH*VGFEVI	PFLQA*DVDDTAK*GLISL	VISFLNFQA*DEFME*ATAGPA
E. amarillans_a	RHQLLEW*DVAATTH*VGFEII	PHLPT*DLDDTAK*GIVSL	IISFLNYQT*DEIME*VVAGAA
E. typhina_a	HHQLLEW*DVAAATH*VGFEII	PHLPT*DLDDTAK*GIVCL	IISFLNYQT*DEFME*VVAGAA
P. amygdali_a	QRQLSDW*DIASTAH*VGFEII	RSISS*DADDTAK*AAFAL	MVSVVDYQA*DEFME*AVAGLT
M. oryzae_a	ERKLSCW*NVSETTH*VGFEII	PSIMA*DADDTAK*TLSAL	HISMLSYQG*DEYME*SVAAPA
P. betae_a	HSLLEEW*DVSTTNN*VGFEII	SGIEP*DVDDSAK*IVTTL	ALSFFTYQV*DEFME*AVAGPA
A. otae_a	KDQLEAW*DVASADR*VGFEIL	CNLEA*DADDTAK*ALLAL	ILSFLNYQA*DEYME*AVVGRY
A. nidulans_c	SRQLQAW*SVKDTVH*VGFEII	PSIQA*DADDTAK*SLIAV	VISFLNYQA*DEFME*AVVGRL
N. fischeri_a	SRELQAW*DVKDTVH*VGFEMI	PSIQA*DADDTAK*ALMTV	VISFLNYQA*DEFME*AVVGRL
T. stipitatus_a	QAKLETW*DVEATDH*VGSEIL	SGVIA*DADDTAR*IILTL	MVSMLNFQV*DRFIE*AVIGQE
P. strigosozonata_a	NTALQTW*DVKSTER*IALEMI	ANVGP*DSDDTAK*ALTAL	VISMVNFQV*DEFFD*IVVQKH
S. lacrymans_a	VDALQGW*DILSTER*IAFEMI	ANVGP*DSDDTAK*ALTAL	VISMVNFQV*DEFFD*LVVQKH
P. anserina_a	RELKAFW*LDATTLP*VGFEML	*DDTAK*GITTL	VISALAYQV*DEFIE*TSVSQL
G. luxurians_a	ASQLNSW*DVSKAER*VGFEVTI	MDTGE*DADDSSK*VLTAL	NMCLVTFQI*DEFFD*SVVQVQ
G. luxurians_b	ASQLNSW*DVSKAER*VGFELTI	LDVGE*DADDSAK*AITAL	AITLINFQV*DEFFD*SMVQDQ
G. luxurians_c	ASQLNSW*DVSKAER*VGFEVTI	MDTGE*DADDSSK*VLTAL	NMCLVTFQI*DEFFD*SVVQVQ
L. palustris_a	ERKLEHW*DPSACDS*VGFEVL	AGMMH*DADDTAK*ALQSL	VISMLNFQA*DAYIE*KVVGKK
P. teres_a	DRQLNAW*DVTLTDH*VGFEII	PYFAP*DVDDTSK*TITSL	TIAMLNFQL*DEFME*ATAGPA
A. nidulans_a	QRLLEAW*DLDGTDQ*VGFEVI	PGILA*DADDTAR*VLLTL	HFSLLVYQV*DALME*STAIRM
A. niger_a	QQALQQW*DVASTLQ*VGSEVL	PGFVP*DADDTAR*ALLTQ	VISLLNYQV*DEYME*SVVALL
A. sojae_a	SDALNSW*NVEATLH*VGFEIL	PGFVP*DADDTAR*SLLAL	VISMLNYQA*DEYME*SVVGSL
T. reesei_a	QSALNNW*DVNQTLH*VGFEML	PTLLP*DADDTAV*SLLAM	VISMLNYQV*DEYME*SVVAYL
R. rufulum_b	NRMLKEW*NIGSTDR*VGFEVL	PSCLP*DADDTAK*TLSAL	IISMLGFQV*DAYME*TVVAEH
G. lozoyensis_a	NHMLQNW*DIKGSDR*VGFEVL	SNFLP*DADDTAK*ANTVL	VVSMLDYQV*DGYME*FVLQEK
A. fumigatus_a	QTALKGL*DRLLATC*TLSVGL	PNACA*DADDTAK*ALVAL	ELSIGLFQE*DDLME*RSLASL
A. oryzae_a	DAALKGL*NGLLRTC*TLPVSL	PKACP*DADDTAK*ALAAL	ELSIGIFQE*DELME*KSLVNL
A. sojae_b	DTALKGL*NDLLATC*TLPVSV	PKACP*DADDTAK*ALAAL	ELSIGIFQE*DELME*KSLVNL
A. nidulans_b	DAALVRL*DDLLATS*TLTVGL	PKACP*DADDTAK*ALIAF	ELSVGIFQE*DEEME*KSLVNL
P. marneffei_a	QKLLDEW*NVDSTLH*VGFEIL	PFVGA*DADDTAT*TILVL	SISIHTDHS*GSYYH*RSDWTT
T. stipitatus_b	QKLLDDW*DVKSTLH*VGFEIL	PFVGA*DADDTAT*TILVL	CVSIHTDDS*DSYYQ*RSQWTI
C. purpurea_a		APHTA*DVDDTAK*AILAL	MLSLLGYQT*DEFIE*AVAGPA
M. fijiensis_a	TTQLRHC*DIESTNH*IGVELV	GQGAT*DADDTAK*GLLAL	FMTLINIQV*DEFIE*AVITPT
C. globosum_a	KEMLHGW*DVESTDQ*VGFEIL	PGSLP*DVDDTAK*GIEAL	VLSVLNFHA*DEYME*GVVEKH
N. crassa_a	QSMLLHW*DVATTDQ*VGFELL	PGGLV*DADDTAR*AIMAL	VLSMLNYQV*DEYME*TAVERD
N. tetrasperma_a	QSMLLTW*DVATTDQ*VGFELL	PGGLV*DADDTAR*AIMAL	VLSMLNYQV*DEYME*TAVERD
S. macrospora_a	KEMLNKW*DVASTDQ*VGFELL	PGGLV*DADDTAR*AIMAL	ILSLLNYQV*DEYME*TAVERD
R. rufulum_a	RYLLGCW*DASKATP*NDLEIL	PGSLQ*DARYIPH*AILAL	YNDYGSATR*DVEEG*NLNSLD
B. fuckeliana_a		PGVLPDVDDTSKGLEAL	VLSILNFHADEYMEGIIERH
X. parietina_a	RRMLSSW*DATSSDD*VGFEVL	PSVLD*ESDDTGS*SIYIL	VFSALLYDF*DHYME*DIIAGF
Z. passerinii_a	ASQLASW*NDVEITN*HIGVEL	PACSR*TMMDQHA*NGSWE	YLIRDHSST*MNSRD*SA

The number of fungal di-TPS genes per genome ranges from one copy (39 species out of 48) in most cases, to two copies (for *C. purpurea*) or paralogs (for *A. sojae*, *M. oryzae*, *P. amygdali*, *R. rufulum*, *T. stipitatus*), or three copies (for *G. luxurians*) or paralogs (for *A. nidulans*). When the phylogenetic position of each of these sequences is examined, two types of clustering are found. 1) in the case of *C. purpurea*, both copies are clustered together, suggesting that they have resulted from a recent duplication event that took place specifically in the genome of this species. In the case of *G. luxurians*, the three copies are clustered together. However, the fact that only five Basidiomycota sequences are available does not show that they resulted from a duplication event that took place specifically in the genome of this species; 2) in all other cases, both paralogs are scattered throughout the tree, showing that it is not straightforward to determine the origin of the additional sequence. However, inter-species clustering of the di-TPS genes can be observed for *A. nidulans* and *A. sojae*, since the di-TPS genes A. nidulans_a and A. sojae_a belong to the same clade, whereas the other two sequences, A. nidulans_b and A. sojae_b, are both located in a different clade.

The fungal di-TPSs also exhibit considerable variation in their intron/exon patterns (Additional file 2). Some species (*A. sojae, A. niger*) have intronless di-TPS but also contain di-TPS genes with introns. There appears to be no unique intron/exon pattern within each of the Ascomycota, Eurotiomycetes, Dothideomycetes and Sordariomycetes groups. On the other hand, the two Basidiomycota *P. strigosozonata* and *S. lacrymans* di-TPSs exhibit a rather similar intron/exon pattern.

The general genomic context in the chromosomic region flanking the fungal di-TPS genes was investigated by searching for potential GGPPS or cytochrome P450 (P450) genes that could create a functional cluster (Additional file 3). Table 2 indicates that 19 out of 56 regions contain a putative GGPPS. The frequency of GGPPS genes per genome is not uniform in the three Ascomycota groups represented by more than one species: the Eurotiomycetes (4/18) have a lower frequency of GGPPSs than the Dothideomycetes (4/11) and Sordariomycetes (8/19). The two Basidiomycota *P. strigosozonata* and *S. lacrymans* di-TPSs-surrounding regions contain a putative GGPPS, whereas no putative GGPPS were found for the three *G. luxurians* di-TPSs-surrounding regions. Table 2 also indicates that P450 are more frequent and numerous than GGPPSs in Ascomycota: only five regions have no P450 (none of these 5 regions without P450 contains a GGPPS), 11 regions have one P450, 17 regions have two P450, 12 regions have three P450, seven regions have four P450, and one region has five P450. The two Basidiomycota *P. strigosozonata* and *S. lacrymans* di-TPS surrounding regions contain six and five putative P450, respectively, whereas no putative P450 were found for the three *G. luxurians* di-TPSs surrounding regions. Although some data related to the di-TPS or its immediate surrounding was available, no information could be found concerning the genomic context of L. palustris_a and C. paspali_a. The four positive hits resulting from a BLAST search across fungal species that were too small to be shortlisted did not contain GPPS or P450 genes next to the putative di-TPS (*A. kawachii* KAW_01797, *A. niger* XP_00139461, *P. nodorum* XP_001792462, *P. strigosozonata* 12-scaffold, *R. rufulum* NODE_501). The *F. oxysporum* EGU79219, considered too large to correspond to a classical di-TPS, forms a putative cluster with one GGPPS and 4 putative P450 genes.

**Table 2:** Fungal specie lifestyles and number of GGPPS and P450 genes clustered next to each di-TPS

Fungus		di-TPS	GGPPS	P450
Agaricomycetes	*Serpula lacrymans var. lacrymans S7.3* ^b^	S. lacrymans_a	1	4
	*Punctularia strigosozonata* ^b^	P. strigosozonata_a	1	3
Lecaronomycètes	*Xanthoria parietina* ^c^	X. parietina_a	1	3
Dothideomycetes	*Rhytidhysteron rufulum* ^a,b^	R. rufulum_a	0	0
	*Rhytidhysteron rufulum* ^a,b^	R. rufulum_b	0	2
	*Pyrenophora teres f. teres 0–1* ^a^	P. teres_a	1	4
	*Bipolaris sorokiniana ND90Pr* ^a^	B. sorokiniana_a	0	2
	*Bipolaris maydis C5* ^a^	B. maydis_a	0	3
	*Phoma betae* ^a^	P. betae_a	1	2
	*Phaeosphaeria nodorum SN15* ^a^	P. nodorum_a	0	1
	*Mycosphaerella fijiensis* ^a^	M. fijiensis_a	0	0
	*Mycosphaerella graminicola IPO323* ^a^	M. graminicola_a	1	3
	*Zymoseptoria passerinii SP63* ^a^	Z. passerinii_a	0	0
	*Sphaceloma manihoticola* ^a^	S. manihoticola_a	1	3
Leotiomycetes	*Botryotinia fuckeliana B05.10* ^a^	B. fuckeliana_a	0	2
	*Glarea lozoyensis ATCC 20868* ^b^	G. lozoyensis_a	0	2
Eurotiomycetes	*Aspergillus fumigatus A1163* ^b,d^	A. fumigatus_a	0	1
	*Aspergillus nidulans FGSC A4* ^b^	A. nidulans_a	0	2
	*Aspergillus nidulans FGSC A4* ^b^	A. nidulans_b	0	1
	*Aspergillus nidulans FGSC A4* ^b^	A. nidulans_c	1	2
	*Aspergillus niger CBS 513.88* ^b,d^	A. niger_a	0	2
	*Aspergillus oryzae RIB40* ^b^	A. oryzae_a	1	1
	*Aspergillus sojae NBRC 4239* ^b^	A. sojae_a	1	2
	*Aspergillus sojae NBRC 4239* ^b^	A. sojae_b	1	1
	*Neosartorya fischeri NRRL 181* ^b,d^	N. fischeri_a	0	0
	*Talaromyces stipitatus ATCC 10500* ^b^	T. stipitatus_a	0	2
	*Talaromyces stipitatus ATCC 10500* ^b^	T. stipitatus_b	0	3
	*Penicillium marneffei ATCC 1822* ^b,d^	P. marneffei_a	0	3
	*Arthroderma benhamiae CBS 112371* ^d^	A. benhamiae_a	0	4
	*Arthroderma otae CBS 113480* ^d^	A. otae_a	0	4
	*Trichophyton equinum CBS 127.97* ^b,d^	T. equinum_a	0	2
	*Trichophyton rubrum CBS 118892* ^b,d^	T. rubrum_a	0	3
	*Trichophyton tonsurans CBS 112818* ^b,d^	T. tonsurans_a	0	2
	*Trichophyton verrucosum HKI 0517* ^b,d^	T. verrucosum_a	0	3
Sordariomycetes	*Phomopsis amygdali* ^a^	P. amygdali_a	1	5
	*Phomopsis amygdali* ^a^	P. amygdali_b	1	1
	*Magnaporthe oryzae 70–15* ^a^	M. oryzae_a	1	1
	*Magnaporthe oryzae 70–15* ^a^	M. oryzae_b	0	2
	*Trichoderma reesei QM6a* ^b^	T. reesei_a	0	2
	*Fusarium fujikuroi* ^a^	F. fujikuroi_a	1	4
	*Fusarium proliferatum* ^a^	F. proliferatum _a	1	4
	*Aciculosporium take* ^a^	A. take_a	0	0
	*Claviceps purpurea 20.1* ^a^	C. purpurea_a	0	3
	*Claviceps purpurea 20.1* ^a^	C. purpurea_b	0	3
	*Epichloe amarillans E57* ^e^	E. amarillans_a	1	2
	*Epichloe typhina E5819* ^e^	E. typhina_a	1	2
	*Podospora anserina S mat+* ^b^	P. anserina_a	0	2
	*Neurospora crassa OR74A* ^b^	N. crassa_a	0	1
	*Neurospora tetrasperma* ^b^	N. tetrasperma_a	0	1
	*Sordaria macrospora k-hell* ^b^	S. macrospora_a	0	1
	*Chaetomium globosum CBS 148.51* ^b,d^	C. globosum_a	0	3
	*Eutypa lata* ^a^	E. lata_a	0	1

In superscript: plant parasites^a^, saprophytes^b^, lichens^c^, animal parasites^d^, plant symbionts^e^

### Horizontal gene transfers

**Fig. 3:**
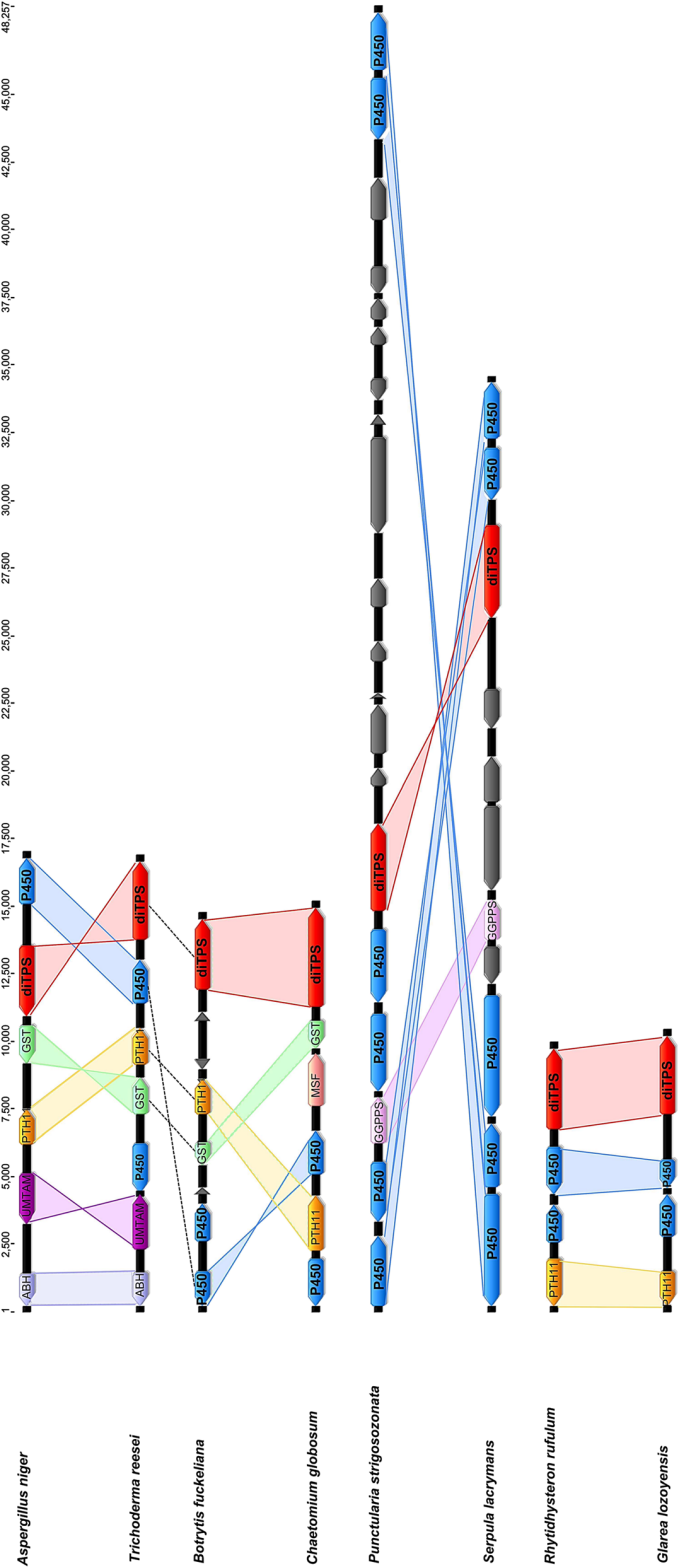
Comparison of gene content and organization in putative diterpene biosynthetic gene clusters, assumed to have undergone HGT between fungi for A. niger_a and T. reesei_a, P. strigosozonata_a and S. lacrymans_a, A. nidulans_a and C. globosum_a, R. rufulum_b and G. lozoyensis_a. The direction and relative size of genes are indicated by arrows; the upper scale indicates the cluster size in bp. The function of putative syntenic genes is: ABH (Alpha/beta hydrolase), di-TPS (diterpene synthase), GGPPS (geranylgeranyl diphosphate synthase), GST (glutathione S-transferase), MSF (major facilitator superfamily transporter), PHT11 (integral membrane protein PTH11-like protein), P450 (Cytochrome P450), UMTAM (S-adenosylmethionine-dependent methyltransferase)

**Fig. 4:**
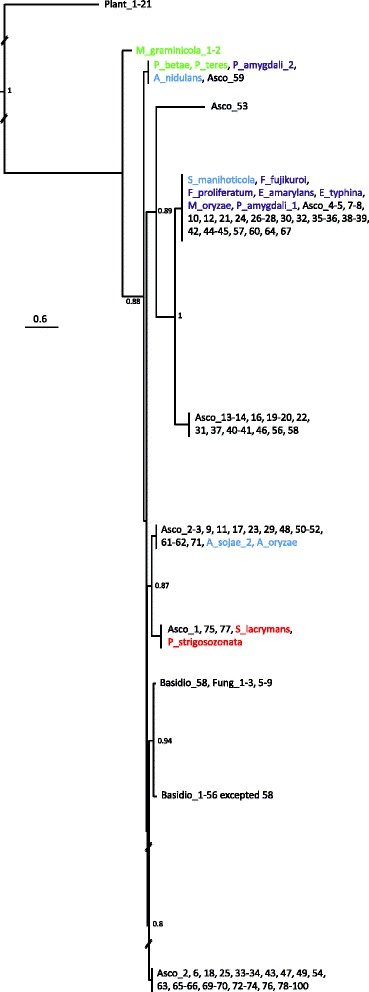
Phylogenetic tree performed on GGPPS. The fungal and plant GGPPS used are listed in Additional file 8. GGPPS not in di-TPS clusters are black in colour. GGPPS in di-TPS clusters are in red for Basidiomycota, blue for Eurotiomycetes, green for Dothideomycetes and purple for Sordariomycetes. The tree is rooted at the level of the plant clade and sequences belonging to the same monophyletic group are clustered together. The branch lengths that had to be reduced for the readability of the figure are striped. The entire phylogenetic tree is described in Additional file 9

Potential di-TPS HGTs between fungi were assessed by visual inspection of the phylogeny and further investigation of the surrounding regions, in order to highlight potential similarities in gene organization between species that are not closely related (Fig. 3). A potential HGT was then confirmed by best hit BLAST searches performed on the genes involved in a potential gene cluster (Additional files 4, 5, 6 and 7). The following set of di-TPSs having similar genes in not closely related organisms was found: A. niger_a and T. reesei_a, C. globosum_a and B. fuckeliana_a, R. rufulum_b and G. lozoyensis_a, S. lacrymans_a and P. strigosozonata_a. Another potential HGT shown in Fig. 2 (P. teres_a and P. amygdali_b, aLRT = 1.0) is not shown in Fig. 3, since no surrounding genes other than GGPPS and P450 were involved in the potential cluster. In the case of the putative *G. lozoyensis* / *R. rufulum* HGT (aLRT = 0.99), it is noteworthy that neither di-TPSs R. rufulum_b, nor G. lozoyensis_a contain the Mg^2+^-ionization/cyclization site encountered in almost all fungal di-TPS. A. niger_a, T. reesei_a, C. globosum_a and A. nidulans_a have a rather similar gene organization, which is highlighted in Fig. 3. The *S. lacrymans* and *P. strigosozonata* putative HGT was further analyzed at GGPPS level, and the GGPPS phylogenetic tree (Fig. 4) shows that *S. lacrymans* EGO03064 and *P. strigosozonata* EIN09901 are not located within the main clade of Basidiomycota GGPPSs, but within a group of 18 Ascomycota GGPPs. The fungal and plant GGPPS used are listed in Additional file 8 and the entire phylogenetic tree is described in Additional file 9. These Ascomycota species correspond to 11 Eurotiomycetes, 4 Sordariomycetes and 3 Saccharomycetes GGPPs. On the other hand, the *S. lacrymans* and *P. strigosozonata* GGPPSs located outside the di-TPS/GGPPS/P450 cluster (Basidio_63, Basidio_64, Basidio_65) do branch with the main clade of Basidiomycota GGPPSs.

## Discussion

One first question that can be raised concerning an HGT is the time of the transfer and its potential relationship with the radiation of the main fungal lineages. Land plants and fungi diverged long ago and display major metabolic and developmental differences. It is also quite speculative to draw an analogy between plant and fungal evolution, because the origin of fungi and the dating of fungal divergences are highly inconsistent. Some of the difficulties arise from the use of a unique value for the molecular clock, and tying the dating of all tree branches to just one dating event in the fossil record. Studies using several fossils to multi-calibrate the clock tend to favour the origin of fungi around 760 to 1060 million years ago (mya) [35, 36]. Other critical dating suggested by these studies include the diversification of Pezizomycotina at around 520–320 mya, thus coincident with the diversification of land plants (480–435 mya) and the assumed mutualistic associations between Ascomycota and plants [37]. Indeed, it is often suggested that the diversification of both the plant and fungal kingdoms in terrestrial environments was partly dependent on their ancient ecological associations [38–40]. However, the exchange of genetic material between both kingdoms has not been widely studied. The data from Figs. 1 and 2 suggest that the di-TPS HGT may have taken place in an ongoing context of complex mutualistic interactions, involving numerous associations such as mycorrhiza, plant diseases, saprotrophic degradation of plant by fungi and commensal interactions.

The study of Richards et al. [10] highlights five HGT events from fungi to plants and four from plants to fungi. This is of particular interest, as it shows that, on several occasions, barriers of the cell walls as well as incorrect intron splicing between unrelated eukaryotes have been overcome. These cross-species events between plants and fungi occurred at different times during evolution, some of them between basal lineages (prior to fungal *Dikarya* and at the beginning of land plants), and others more recently (during early diversification of Pezizomycotina and following bryophyte differentiation). The number of proposed HGT is clearly incorrectly estimated, since their identification depends not only on the stringency of the method used but also on subsequent events. For example, HGT can occasionally be overestimated due to lineage specific gene loss, as highlighted by the case of the pleiotropic drug resistance ABC transporter family that is found in plants and fungi, but is absent in animals. These are thought to have been present in the ancestors of all three kingdoms, but then to have been lost in the animal lineage. It is thus likely that the ABC transporter genes were not transferred horizontally between plants and fungi [41]. In our study, similarity searches using di-TPSs show that, until now, di-TPS genes have not been detected in most eukaryotic genomes, with the notable exception of plants and fungi. In addition, all higher plants have gibberellins, whereas only a few fungi appear to contain di-TPS genes. This strongly suggests that the fungal di-TPSs we studied were not present in the ancestor of eukaryotes and later lost in most fungal lineages but that they were actively acquired by HGT from other organisms such as plants. The fact that: 1) only a few fungal species contain di-TPSs (Fig. 1); 2) some species contain several di-TPSs (Table 2); 3) some of these di-TPSs are intronless (Additional file 2); and 4) the phylogenetic relationships of fungal di-TPPs are not in agreement with inferred phylogenetic relationships between species and higher order lineages of fungi (Figs. 1 and 2), indicates that since the original HGT, these genes have been actively transferred and duplicated and have evolved in fungi.

One could therefore ask whether the plant di-TPSs originally transferred to fungi as a functional di-TPS/GGPPS/P450 gene cluster encoding an end product or whether the selection for production of the diterpene secondary metabolites resulted in the formation of clusters comprising di-TPS, GGPPS and P450 genes. Until recently, it was thought that genes for plant metabolic pathways were not clustered, and it would have been logical to exclude the possibility of a di-TPS cluster HGT from plants to fungi. However, five clusters of genes for plant metabolic pathways have recently been discovered, and all of these are implicated in the synthesis of defence compounds [42]. Two of these clusters, the rice momilactone and phytocassane clusters, even correspond to diterpene biosynthesis genes [43]. The possibility of a past di-TPS cluster HGT should thus be discussed in this context. Part of this scenario can be answered by considering the GGPPS genes found in fungi containing di-TPS. Figure 4 shows that these genes have more similarities to other fungal GGPPSs and that they are not closely related to plant GGPPSs. Another component of this scenario can be found by considering the well-studied gibberellic acid (GA) biosynthetic pathway as a model. In land plants, this pathway is based on further modifications by P450s of *ent*-kaurene produced by the di-TPS [20, 44]. Plant P450s catalyse a wide variety of monooxygenation/hydroxylation reactions in primary and secondary metabolisms, including GA biosynthesis. Plant genomes contain a large number of P450, and it is estimated that they account for approximately 1 % of genes [44, 45]. It is noteworthy that P450 fungal genes are not closely related to plant P450 genes, since they share only 10-15 % of the amino-acid identity [29]. It is also noteworthy that the GA biosynthetic genes are clustered on a single chromosome in fungi, whereas they are randomly located on multiple chromosomes in plants [20, 44]. Furthermore, the intermediates in GA biosynthesis are different in plants and fungi. This strongly suggests that plants and fungi have evolved their complex GA biosynthesis pathways independently [29] and that di-TPSs were not acquired from the plants as a whole cluster set.

The discovery of most of the di-TPS fungal genes in clusters with P450 and, to a lesser extent, GGPPSs, is not surprising, since a notable characteristic of fungal genomes is that genes involved in successive steps of a metabolic pathway are often physically linked or clustered [46]. However, in the present study Table 2 shows that in Ascomycota these genes are not clustered with a unique organization, suggesting several mobilization modalities of P450 and GGPPS genes following insertion of the di-TPS gene in the fungal genome. A similar event has already been described in fungi, such as in the case of the galactose utilization pathway. It has been demonstrated that in two cases, fungal galactose gene clusters originated independently (once within Ascomycota and once within Basidiomycota) [47].

The presence of di-TPSs in Basidiomycota was not anticipated, since the literature indicates that they appear to be present only in some branches of the Ascomycota. The similarity searches and the phylogeny based on Basidiomycota GGPPS sequences clearly show that these genes share a closer similarity with Ascomycota than with plant GGPPS sequences, suggesting a fungal origin for these di-TPS. The analysis of the genomic context of the di-TPSs in *S. lacrymans* and *P. strigosozonata* highlighted a putative cluster containing one GGPPS and several P450 genes. A comparison between *S. lacrymans* and *P. strigosozonata* genes shows a rather high degree of similarity between both clusters, with the respective ortholog sequences from *S. lacrymans* and *P. strigosozonata* clusters usually beeing the first to occur in similarity searches. This cluster was found in *P. strigosozonata* in a more compacted form than in *S. lacrymans*. The GGPPS tree shows that the GGPPS genes from *S. lacrymans* and *P. strigosozonata* clusters branch with none of the Basidiomycota GGPPSs, contrary to the other three GGPPS genes found in *S. lacrymans* and *P. strigosozonata* genomes. This strongly suggests a common, or at least similar, origin for both di-TPS/GGPPS/P450 clusters found in two distantly related Basidiomycota species. It is therefore possible to speculate that the di-TPS genes may have been transferred from Ascomycota to Basidiomycota. An Ascomycota pathogen infecting a wide array of other fungal species appears to be the easiest way to imagine such a gene transfer. There are examples of such mycoparasites in each of the main Ascomycota groups found to contain di-TPS in our study, for example, *Gliocladium roseum*, *Trichoderma sp.* (Sordariomycetes), *Penicillium restrictum* (Eurothiomycetes) [48–50].

It was however surprising to find that the different di-TPS sequences did not group in the tree according to the fungal phylogeny (Lecaronomycetes, Dothideomycetes, Leotiomycetes, Eurotiomycetes, Sordariomycetes) but rather exhibited some similarities depending on lifestyle, with a group comprising mainly plant-associated fungi (pathogens or symbionts) (Fig. 2). On the other hand, sequences from saprophytic and animal pathogenic fungi are scattered into three different paraphyletic groups, and no clear trend can be identified at this level.

Gibberellins have been extensively studied in *F. fujikuroi* since 1926, and this fungus can be considered as a model of a fungal pathogen using a diterpene hormone. A functional gibberellin synthase cluster contains seven genes encoding: a bifunctional *ent*-copalyl and *ent-*kaurene diphosphate synthase, a geranylgeranyl diphosphate synthase, four P450 monooxygenases and a desaturase [51, 52]. Although gibberellin synthase cluster regulation is linked to the nitrogen metabolism and the enhancement of fungal growth, this can also have an impact on infection efficiency. This production of gibberellin induces abnormal elongation of plants, early dry up of the leaves, lesions on the roots and adventitious roots, and only partially filled, sterile, or empty seeds. Several other fungi belonging to Ascomycota and Basidiomycota have since been identified as able to produce gibberellins involved in pathogenic or, more interestingly, plant growth promoting interactions [29]. Also, our study often detected di-TPS genes in plant fungal pathogens (Fig. 1) that belong not only to the Ascomycota (Leotiomycetes, Sordariomycetes and Dothideomycetes) but also to the Basidiomycota (*M. perniciosa*).

Surprisingly, the potentially hormonal role of fungal diterpenes in a symbiotic or endophytic fungal/plant relationship [53–55], as well as their antimicrobial properties against bacteria and other fungi [56, 57], have rarely been described in the literature. This trend was confirmed in the present study, since a search for di-TPS-containing fungi found only *Xanthoria parietina, Fusarium proliferatum, Epichloe typhina* and *E. amarillans* as possible examples of symbionts or endophytes. *X. parietina* does not interact with a vascular plant, but lives as a lichen with green algae belonging to the genus *Trebouxia. F. proliferatum* is a plant pathogen, although some strains are mutualistic with orchids [58], *E. typhina* and *E. amarillans* produce the choke disease in grasses, but are also considered as endophytes.

It is noteworthy that diterpenes also appear to have found a hormonal role in fungal development, outside the context of a plant/fungal interaction, since several fungi belonging to the Eurotiales, exhibit conidia and cleistothecia formation linked to diterpene synthesis [41, 59, 60]. The saprophyte fungus *Aspergillus nidulans* contains two functional gene clusters involved in the synthesis of the diterpenoid metabolite *ent*-pimara-8(14),15-diene. Overproduction of this metabolite affects the formation of conidia (decrease in number) and cleistothecia (increase in number) [41, 59]. This cluster of eight co-regulated genes identified by Bromann et al. [59] contains a GGPPS, an HMG-CoA reductase, a di-TPS, a translation elongation factor ©, a short-chain dehydrogenase, a hypothetical protein somewhat similar to methyltransferase, a cytochrome P450, and a Zn(II)_2_Cys_6_-type transcription factor. We found a very similar cluster for the di-TPS containing Eurotiales *Neosartorya fischeri* but not for other Eurotiales (*A. fumigatus, A. niger, A. oryzae, A. sojae, Talaromyces stipitatus, Penicillium marneffei*). Furthermore, di-TPSs in the latter Eurotiales species did not group in the phylogenetic tree with *A. nidulans* and *N. fischeri* di-TPSs (Fig. 2), thus indicating that they are not as closely related as the enzyme identified by Bromann et al. [59].

Not all di-TPSs synthesize diterpenes with hormonal activity. Toxic diterpenes have also been described in fungi with various lifestyles (plant or animal parasites, endophytes, or saprophytes) and in plants. Fungal toxic diterpenes include various diterpenes such as indole diterpenes (paspaline, paxillin, shearinines, paspalitrems, terpendoles, penitrems, lolitrems, janthitrems, and sulpinines [61–63]), aphidicolins, and fusicoccins. Each species can produce a family of diterpenes with various toxic effects, such as neurotoxins acting: through modulation of the central nervous system and inhibition of the potassium channel in the peripheral nervous system (paxilline from *Penicillium paxilli*, lolitrems from *Neotyphodium lolii,* aflatrems from *Aspergillus flavus*), as an inhibitor of DNA polymerase 〈 (aphidicolin from *Phoma betae* [60, 64]), and as an activator of Membrane H^+^-ATPase from higher plants (methyl phomopsenonate and phytotoxin fusicoccins from *Phomopsis amygdali* [65, 66]).

So far, diterpenes have been described as acting either as hormones, or as toxic molecules. In the present study, fungal di-TPSs have been found in species with lifestyles (plant or animal parasites, saprophytes, and symbionts) suiting both of these biological functions. The good conservation of Mg^2+^-PPi binding, H^+^-initiated cyclization and Mg^2+^-ionization/cyclization also suggests that these enzymes are functional in the fungal cell. It is therefore expected that each of the di-TPS genes we studied has been under pressure to evolve in such a way as to better accommodate one of these functions, which could explain why our di-TPS tree (Fig. 2) does not reflect the fungal phylogenetic position but rather the fungal lifestyle. This function-related tree also suggests that in general, when fungi have no di-TPS in their genomes, this is not the result of the loss of an original di-TPS gene. It is however noteworthy that there is evidence that gene loss can also occasionally contribute to this distribution [67]. Fungal species with di-TPS genes are more likely to have obtained their di-TPS gene(s) by an ongoing HGT process, occuring in fungi. One approach to the investigation of this possibility involved searching for gene synteny in unrelated species, resolved within the same clade in the di-TPS tree. The di-TPS tree was indeed found to contain examples of such a potential HGT.

## Conclusions

The analysis of fungal di-TPS traced the evolutionary history of an ancient HGT event of a single di-TPS gene from a plant to an Ascomycota. Genome analysis demonstrated that in fungi, these di-TPSs allowed for the formation of clusters of di-TPS, GGPPS and P450 genes that presumably originated independently and were characterized by different mobilization modalities. We observed that members of these clusters can also be transferred between *Dikarya* fungal species. Our analysis leads to a better understanding of how trabfers of di-TPS have enabled fungi to produce diterpenes acting as hormones or toxins. The variable presence of these compounds has implications for the fungal development and pathogenicity.

## Methods

### Sequence database searches

We used BLAST (BLASTP, TBLASTN and PSI-BLAST [68]) and PipeAlign [69] searches to recover information related to the presence or absence of putative di-TPS genes in the available fungal genomes. We started with the sequence from *Fusarium fujikoroi* as a seed and extended our search with di-TPS sequences from each fungal class using an expectation value threshold of E ≤ 10^−6^*.* Searches were performed in the NCBI (http://www.ncbi.nlm.nih.gov/), UniProtKB (http://www.uniprot.org/), Genoscope (http://www.genoscope.cns.fr/spip/) and JGI (http://www.jgi.doe.gov/) collections [date last accessed 25 January 2014], until no new sequences were found (Additional file 1). Sequences encoding incomplete di-TPSs were discarded. Sesquiterpenoid trichothecene synthase-like genes found in several fungi and having weak similarities to di-TPSs were also discarded. Synteny conservation was determined by BLASTP and TBLASTN searches (E ≤ 10^−6^), using approximately 10 genes upstream and downstream from the di-TPS genes to find their orthologs. Completed fungal genomes were numbered using NCBI, JGI and 1,000 fungal genome project databases. For every di-TPS gene accession (Additional file 1), the systematic classification found on the associated web page was used for the fungal phylogenetic tree (Fig. 1).

We used the *Saccharomyces cerevisiae* Geranyl Geranyl Diphosphate Synthase (GGPPS) sequence as a seed to search against the NR database (ncbi http://www.ncbi.nlm.nih.gov), in order to recover additional putative fungal GGPPSs. As a consequence of the large number of Ascomycota sequences available, we selected 100 sequences representative of the diversity of Ascomycota, and used all available GGPPS sequences from the other fungal groups, as well as GGPPS from four plant species (Additional file 3). Putative plant GGPPSs were selected from BLAST searches performed against *Physcomitrella patens*, *Selaginella moellendorffii* and *Zea mays* genome sequences using the *Arabidopsis thaliana* sequence [70] as a seed.

### Gene predictions and intron/exon architecture

Absent or improper gene predictions for the following di-TPS sequences were corrected using the fgenesh SoftBerry software [71]: *Punctularia strigosozonata* (5-scaffold join 1540005–1539226; 1539170–1539050; 1538993–1538707; 1538636–15370098; 1537040–1536985; 1536849–1536666), *Rhystidhysteron rufulum* (NODE_2525 join 7075–7866; 7917–8037; 8091–8371; 8426–10078), *R. rufulum* (NODE_28 join 20355–21152; 21200–21589; 22055–22572; 22828–23059; 23574–23893; 23957–24170; 24225–24307; 24382–24475). The exon and intron positions were formatted into a gff file. The domain architectures (Mg^2+^-PPi binding, H^+^-initiated cyclization and Mg^2+^-ionization/cyclization) of all analysed sequences were predicted from a multiple sequence alignment of the selected sequence using multalin [72] and *Abies grandis* abietadiene synthase as references. Geneious 6.1.6 (Biomatters, www.geneious.com) was used to view the gene structure and generate Table 1.

### Phylogenetic analyses

The same general procedure was used for both datasets (di-TPS and GGPPS). Multiple sequence alignments were computed with Muscle [73], with all default parameters. The alignments were then trimmed using Gblocks [74], with the most relaxed set of parameters. The number of sites retained after trimming was equal to 521 for di-TPS and 186 for GGPPS. Before computing the phylogenies a substitution model selection was realized using ProtTest [75] and the Akaike Information Criterion (AIC). The models selected were LG + ℘_4_ + I + F in the case of di-TPS and LG + ℘_4_ in the case of GGPPS. The phylogenies were computed using the SeaView implementation of PhyML [76]. Alpha parameters of the two Gamma distributions, as well as amino acid invariant frequencies and equilibrium frequencies were estimated using PhyML. Branch supports were determined under PhyML with the Shimodaira-Hasegawa (SH) likelihood test. Branch supports were determined under PhyML with the approximate Likelihood Ratio Test (aLRT) non-parametric branch support based on a Shimodaira-Hasegawa-like procedure.

## Additional files

Additional file 1:
**List of di-TPSs for fungi and 11 plant species.** (DOCX 18 kb) Additional file 2:
**Fungal exons (blue) and introns (black) in putative di-TPS genes.** Fungal species are arranged in the same order as in Table 1 and the subgroups are specified in superscript: Agaricomycetes (Ag), Lecaronomycetes (Lecar), Eurotiomycetes (Eu), Leotiomycetes (Leotio), Sordariomycetes (So), Dothideomycetes (Do). (PDF 2136 kb) Additional file 3:
**Biosynthesis of diterpenoids with symbols indicating the chemical products (rectangles), enzymes (shaded rectangles) and types of catalysed reaction (brackets).** (PDF 45 kb) Additional file 4:
**Best BLAST hits for the genes involved in the putative** ***A. niger*** **and** ***T. reesei*** **gene cluster [73].** (DOCX 13 kb) Additional file 5:
**Best BLAST hits for the genes involved in the putative** ***S. lacrymans*** **and** ***P. Strigosozonata*** **gene cluster.** (DOCX 14 kb) Additional file 6:
**Best BLAST hits for the genes involved in the putative** ***B. fuckeliana*** **and** ***C. globosum*** **gene cluster.** (DOCX 15 kb) Additional file 7:
**Best BLAST search performed on the genes involved in the putative** ***R. rufulum*** **and** ***G. lozoyensis*** **gene cluster.** (DOCX 11 kb) Additional file 8:
**List of GGPPSs containing di-TPS Ascomycota and Basidiomycota species, early diverging lineages of fungi, and three plant species.** (DOCX 23 kb) Additional file 9:
**Entire Phylogenetic tree generated with GGPPS.** (PDF 11 kb)
