# Supplementary material for: Molecular and functional evolution of the fungal diterpene synthase genes
Source: BMC Microbiol. 2015 Oct 19;15:221. doi: 10.1186/s12866-015-0564-8 (PMC4617483; doi:10.1186/s12866-015-0564-8)
Supplement: Additional file 1: — List of di-TPSs for fungi and 11 plant species. (DOCX 18 kb) [file 12866_2015_564_MOESM1_ESM.docx]

**Additional file 1**. List of di-TPS for fungi and 11 plant species.

| Sequence name | Organism | Accession |
| --- | --- | --- |
| A. take_a | *Aciculosporium take* | AFQZ01000150 |
| A. benhamiae_a | *Arthroderma benhamiae CBS 112371* | XP_003013365 |
| A. otae_a | *Arthroderma otae CBS 113480* | XP_002849529 |
| A. fumigatus_a | *Aspergillus fumigatus A1163* | EDP52216 |
| A. nidulans_a | *Aspergillus nidulans FGSC A4* | XP_660856 |
| A. nidulans_b | *Aspergillus nidulans FGSC A4* | XP_682583 |
| A. nidulans_c | *Aspergillus nidulans FGSC A4* | XP_659198 |
| A. niger_a | *Aspergillus niger CBS 513.88* | XP_001398730 |
| A. oryzae_a | *Aspergillus oryzae RIB40* | XP_001820661 |
| A. sojae_a | *Aspergillus sojae NBRC 4239* | contig00364 |
| A. sojae_b | *Aspergillus sojae NBRC 4239* | contig00877 |
| B. maydis_a | *Bipolaris maydis C5* | EMD87156 |
| B. sorokiniana_a | *Bipolaris sorokiniana ND90Pr* | EMD58266 |
| B. fuckeliana_a | *Botryotinia fuckeliana B05.10* | BC1G_13295 |
| C. globosum_a | *Chaetomium globosum CBS 148.51* | XP_001227327 |
| C. paspali_a | *Claviceps paspali* | AFRC01000285 |
| C. purpurea_a | *Claviceps purpurea 20.1* | CCE32886 |
| C. purpurea_b | *Claviceps purpurea 20.1* | CCE30817 |
| E. amarillans_a | *Epichloe amarillans E57* | AFRF01000120 |
| E. typhina_a | *Epichloe typhina E5819* | AFSE01000078 |
| E. lata_a | *Eutypa lata* | EMR62953 |
| F. proliferatum_a | *Fusarium proliferatum ET1* | CAP74389 |
| G. fujikuroi_a | *Fusarium fujikuroi* | CPSKS_GIBFU |
| G. lozoyensis_a | *Glarea lozoyensis ATCC 20868* | EPE31716 |
| G. luxurians_a | *Gymnopus luxurians FD-317M1* | KIK63275.1 |
| G. luxurians_b | *Gymnopus luxurians FD-317M1* | KIK57343.1 |
| G. luxurians_c | *Gymnopus luxurians FD-317M1* | KIK57345.1 |
| L. palustris_a | *Lepidopterella palustris* | scaffold_377 |
| M. oryzae_a | *Magnaporthe oryzae 70-15* | MGG_14722.6 |
| M. oryzae_b | *Magnaporthe oryzae 70-15* | MGG_01949.6 |
| M. fijiensis_a | *Mycosphaerella fijiensis* | 6-scaffold |
| M. graminicola_a | *Mycosphaerella graminicola IPO323* | EGP91192 |
| N. fischeri_a | *Neosartorya fischeri NRRL 181* | XP_001264196 |
| N. crassa_a | *Neurospora crassa OR74A* | XP_958491 |
| N. tetrasperma_a | *Neurospora tetrasperma* | EGO54989 |
| P. marneffei_a | *Penicillium marneffei ATCC 1822* | XP_002152916 |
| P. nodorum_a | *Phaeosphaeria nodorum SN15* | CPSKS_PHASA |
| P. betae_a | *Phoma betae* | BAD29971 |
| P. amygdali_a | *Phomopsis amygdali* | BAG30961 |
| P. amygdali_b | *Phomopsis amygdali* | BAG30962 |
| P. anserina_a | *Podospora anserina S mat+* | XP_001904205 |
| P. strigosozonata_a | *Punctularia strigosozonata* | EIN09905 |
| P. teres_a | *Pyrenophora teres f. teres 0-1* | XP_003297768 |
| S. lacrymans_a | *Serpula lacrymans var. lacrymans S7.3* | EGO03067 |
| S. manihoticola_a | *Sphaceloma manihoticola* | CAP07655 |
| R. rufulum_a | *Rhytidhysteron rufulum* | NODE_28 |
| R. rufulum_b | *Rhytidhysteron rufulum* | NODE_2525 |
| S. macrospora_a | *Sordaria macrospora k-hell* | XP_003345649 |
| T. stipitatus_a | *Talaromyces stipitatus ATCC 10500* | EED11984 |
| T. stipitatus_b | *Talaromyces stipitatus ATCC 10500* | EED14438 |
| T. reesei_a | *Trichoderma reesei QM6a* | EGR51467 |
| T. equinum_a | *Trichophyton equinum CBS 127.97* | EGE08989 |
| T. rubrum_a | *Trichophyton rubrum CBS 118892* | XP_003231050 |
| T. tonsurans_a | *Trichophyton tonsurans CBS 112818* | EGD99332 |
| T. verrucosum_a | *Trichophyton verrucosum HKI 0517* | XP_003025434 |
| X. parietina_a | *Xanthoria parietina* | Xp 1-scaffold |
| Z. passerinii_a | *Zymoseptoria passerinii SP63* | AFIY01001118 |
|  |  |  |
| Plant_a | *Physcomitrella patens* | BAF61135 |
| Plant_b | *Selaginella moellendorffii* | XP_002960350 |
| Plant_c | *Oryza sativa Japonica Group* | NP_001046550 |
| Plant_d | *Zea mays* | NP_001105257 |
| Plant_e | *Arabidopsis thaliana* | GA1 |
| Plant_f | *Glycine max* | XP_003520571 |
| Plant_g | *Medicago truncatula* | MTR_3g058160 |
| Plant_h | *Populus trichocarpa* | EEE81383 |
| Plant_i | *Sorghum bicolor* | XP_002464625 |
| Plant_j | *Vitis vinifera* | XP_002272869 |
| Plant_k | *Picea abies* | AAS47691 |
