# Supplementary figures and images for: Molecular and functional evolution of the fungal diterpene synthase genes
Source: BMC Microbiol. 2015 Oct 19;15:221. doi: 10.1186/s12866-015-0564-8 (PMC4617483; doi:10.1186/s12866-015-0564-8)

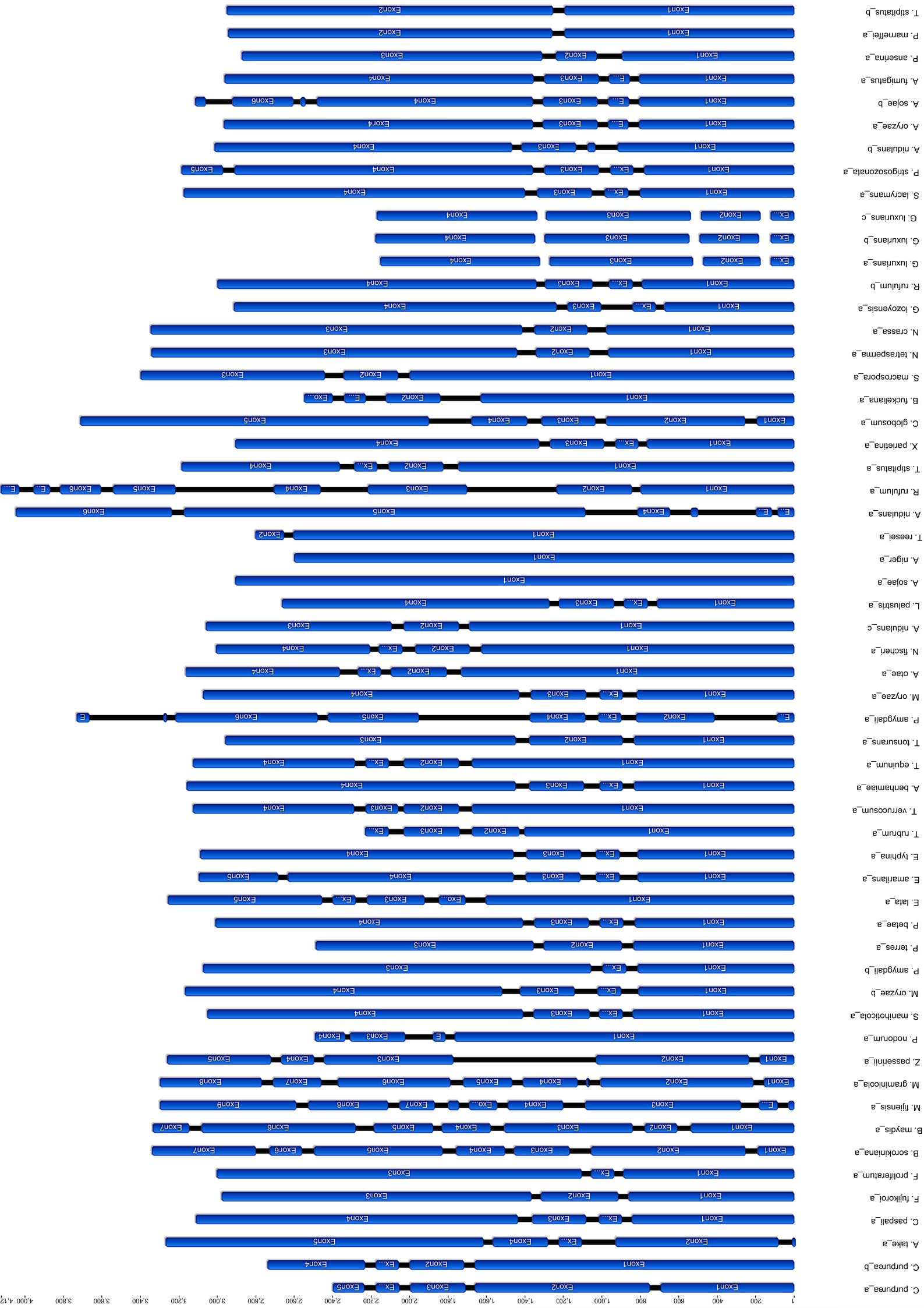

Supplement: Additional file 2: — Fungal exons (blue) and introns (black) in putative di-TPS genes. Fungal species are arranged in the same order as in Table 1 and the subgroups are specified in superscript: Agaricomycetes (Ag), Lecaronomycetes (Lecar), Eurotiomycetes (Eu), Leotiomycetes (Leotio), Sordariomycetes (So), Dothideomycetes (Do). (PDF 2136 kb) [file 12866_2015_564_MOESM2_ESM.pdf]
