# Supplementary material for: Molecular and functional evolution of the fungal diterpene synthase genes
Source: BMC Microbiol. 2015 Oct 19;15:221. doi: 10.1186/s12866-015-0564-8 (PMC4617483; doi:10.1186/s12866-015-0564-8)
Supplement: Additional file 3: — Biosynthesis of diterpenoids with symbols indicating the chemical products (rectangles), enzymes (shaded rectangles) and types of catalysed reaction (brackets). (PDF 45 kb) [file 12866_2015_564_MOESM3_ESM.pdf]

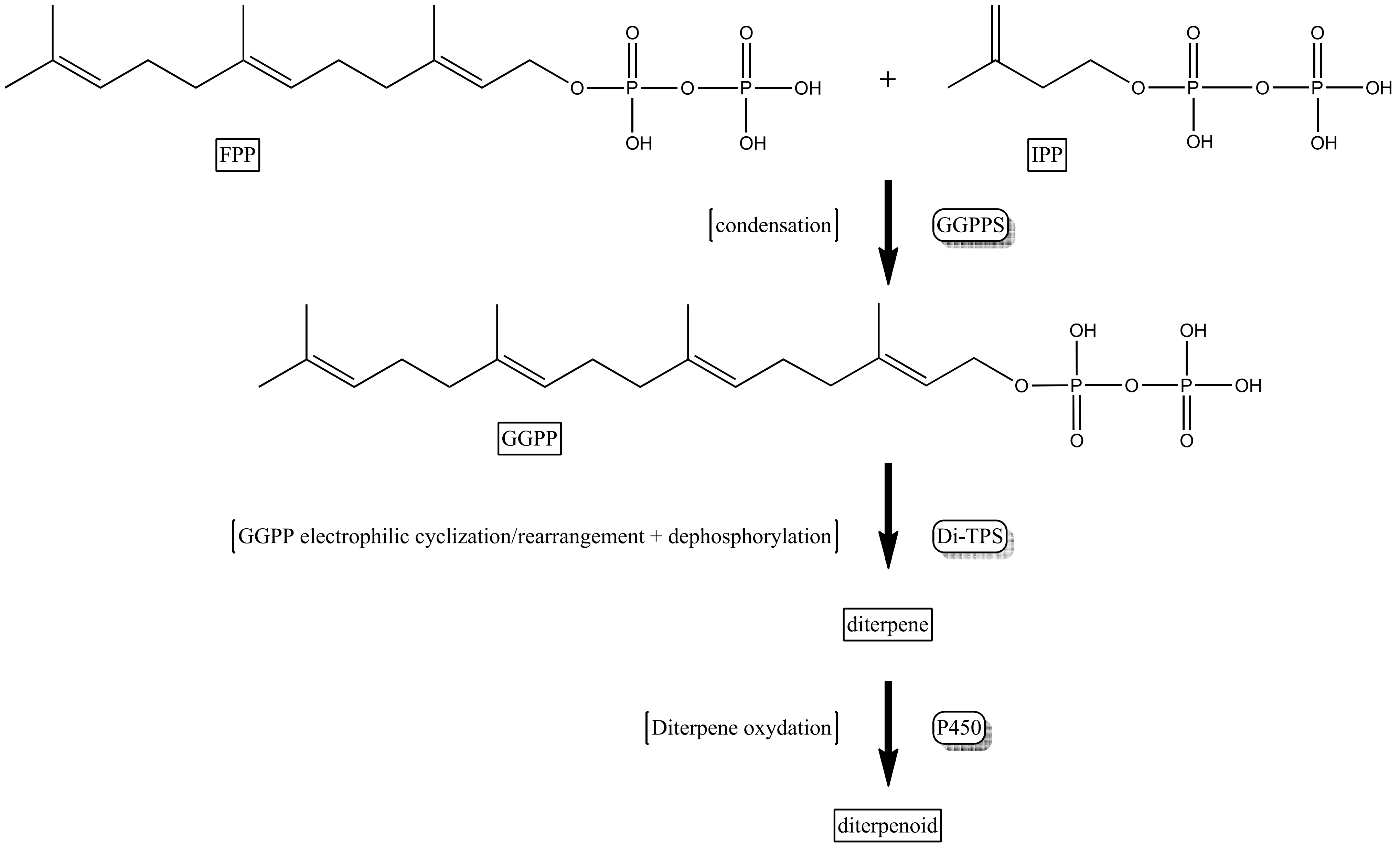

FPP
IPP
condensation
GGPPS
GGPP
GGPP electrophilic cyclization/rearrangement + dephosphorylation
Di-TPS
diterpene
Diterpene oxydation
P450
diterpenoid
