# Supplementary material for: Molecular and functional evolution of the fungal diterpene synthase genes
Source: BMC Microbiol. 2015 Oct 19;15:221. doi: 10.1186/s12866-015-0564-8 (PMC4617483; doi:10.1186/s12866-015-0564-8)
Supplement: Additional file 4: — Best BLAST hits for the genes involved in the putative A. niger and T. reesei gene cluster [73]. (DOCX 13 kb) [file 12866_2015_564_MOESM4_ESM.docx]

**Additional file 4:**  best BLAST hits for the genes involved in the putative *A. niger* and *T. reesei* gene cluster.

| ***A.niger*** | **Best hit^a^** | **2^nd^ Best Hit^a^** | **third Best hit^a^** | **Putative function^b^** |  | ***T. reesei*** | **Best hit^a^** | **2^nd^ Best Hit^a^** | **third Best hit^a^** | **Putative function^b^** |
| --- | --- | --- | --- | --- | --- | --- | --- | --- | --- | --- |
| *Aspergillus niger* XP_001398735.2 | *Trichoderma virens* EKA18944.1 (61%) | *Trichoderma reese*i EGR51187.1 (59%) | *Glaerea lozoyeansis* EPE25884.1 (40%) | ABH |  | *Trichoderma reesei* EGR51187.1 | *Trichoderma virens* EKA18944.1 (73%) | *Aspergillus niger* XP_001398735.2 (57%) | *Glaerea lozoyeansis* EPE25884.1 (38%) | ABH |
| *Aspergillus niger* XP_001398734.2 | *Trichoderma reesei* EGR51470.1 (83%) | *Phaeosphaeria nodorum* XP_001801132.1 (35%) | *Eutypa lata* EMR61345.1 (42%) | UMTAM |  | *Trichoderma reesei* EGR51470.1 | *Aspergillus niger* XP_001398734.1 (33%) | *Aspergillus clavatus* XP_001269247.1 (31%) | *Phaeosphaeria nodorum* XP_001801132.1 (33%) | UMTAM |
| *Aspergillus niger* XP_001398732.2 | *Trichoderma reesei* EGR51469.1 (57%) | *Chaetomium globosum* XP_001227323.1 (32%) | *Aspergillus nidulans* XP_660861.1 (32%) | PTH11 |  | *Trichoderma reesei* EGR51186.1 | *Aspergillus kawachii* GAA83294.1 (53%) | *Aspergillus niger* CAK48657.1 (51%) | *Botrytiana fuckeliana* EMR86103.1 (35%) | P450 |
| *Aspergillus niger* XP_001398731.2 | *Aspergillus kawachii* GAA83293.1 (96%) | *Trichoderma reesei* EGR51185.1 (79%) | *Botrytiana fuckeliana* EMR86102.1 (53%) | GST |  | *Trichoderma reesei* EGR51469.1 | *Aspergillus niger* XP_001398732.2 (51%) | *Phaeospharia nodorum* XP_001801131.1 (33%) | *Chaetomium globosum* XP_001227323.1 (33%) | PTH11 |
| *Aspergillus niger* XP_001398730.2 | *Trichoderma reesei* EGR51467.1 (49%) | *Arthroderma otae* XP_002849529.1 (37%) | *Neosartorya fischeri* XP_001264196.1 (43%) | di-TPS |  | *Trichoderma reesei* EGR51185.1 | *Aspergillus kawachii* GAA83293.1 (78%) | *Aspergillus niger* XP_001398731.2 (78%) | *Botrytiana fuckeliana* EMR86102.1 (52%) | GST |
| *Aspergillus niger* XP_001398728.2 | *Trichoderma reesei* EGR51468.1 (76%) | *Botryotinia fuckeliana* CCD45418.1 (63%) | *Neosartorya fischeri* XP_001264200.1 (43%) | P450 |  | *Trichoderma reesei* EGR51468.1 | *Aspergillus niger* XP_001398729.1 (76%) | *Botryotinia fuckeliana* CCD45418.1 (62%) | *Neosartorya fischeri* XP_001264200.1 (45%) | P450 |
| *Aspergillus niger* XP_001398728.2 | *Talaromyces marneffei* XP_0021456737.1 (57%) | *Aspergillus kawachii* GAA92012.1 (57%) | *Botrytis fuckeliana* CCD46537.1 (54%) | MOX |  | *Trichoderma reesei* EGR51467.1 | *Aspergillus niger* XP_001398730.2 (49%) | *Arthroderma otae* XP_002849529.1 (40%) | *Neosartorya fischeri* XP_001264196.1 (38%) | di-TPS |
| *Aspergillus niger* XP_001398727.2 | *Aspergilus kawachii* GAA83292.1 87% | *Colletotrichum lindemuthianum* ACN71233.1 (37%) | *Sclerotinia borealis* ESZ98215.1 (38%) | P450 |  |  |  |  |  |  |

^a^Homology searches were performed by using BLAST [73]). For each protein of the cluster, the percentage of identity are indicated for the three best hits.

^b^Putative functions : ABH (Alpha/beta hydrolase), UMTAM (S-adenosylmethionine-dependent methyltransferase), PHT11 (integral membrane protein PTH11-like protein), GST (glutathione S-transferase), di-TPS (di terpene synthase), P450 (Cytochrome P450), MOX (Multicopper oxidase).
