# Supplementary material for: Molecular and functional evolution of the fungal diterpene synthase genes
Source: BMC Microbiol. 2015 Oct 19;15:221. doi: 10.1186/s12866-015-0564-8 (PMC4617483; doi:10.1186/s12866-015-0564-8)
Supplement: Additional file 5: — Best BLAST hits for the genes involved in the putative S. lacrymans and P. Strigosozonata gene cluster. (DOCX 14 kb) [file 12866_2015_564_MOESM5_ESM.docx]

**Additional file 5:**  best BLAST best hits for the genes involved in the putative *S. lacrymans* and *P. strigosozonata* gene cluster.

| ***S. lacrymans*** | **Best hit^a^** | **2^nd^ Best Hit^a^** | **third Best hit^a^** | **Putative function^b^** |  | ***P. strigosozonata*** | **Best hit^a^** | **2^nd^ Best Hit^a^** | **third Best hit^a^** | **Putative function^b^** |
| --- | --- | --- | --- | --- | --- | --- | --- | --- | --- | --- |
| EGO03062.1 | *P. strigosozonata* EIN09907.1 (66%) | *Ceriporiopsis subvermispora* EMD31428.1 (29%) | *Ceriporiopsis subvermispora* EMD31509.1 (27%) | P450 |  | EIN09896.1 | *S . lacrymans* EGO03073.1 (62%) | *P. strigosozonata* EIN09907.1 (26%) | *S . lacrymans* EGO03062.1 (25%) | P450 |
| EGO03063.1 | *P. strigosozonata* EIN09906.1 (63%) | *Laccaria bicolor* XP8001885786.1 (33%) | *Rhizoctonia solani* CCO31795.1 (36%) | P450 |  | EIN09897.1 | *S . lacrymans* EGO03072.1 (66%) | *Coniosporium apollinis* EON69905.1 (30%) | *Fusarium oxysporum* EMT74057.1 (29%) | P450 |
| EGO03064.1 | P. strigosozonata EIN09901.1 (60%) | *Capsaspora owcarzaki* XP 004363786.1 (46%) | *Rhizopus delemar* EIE80306.1 (44%) | GGPPS |  | EIN09899.1 | *S . lacrymans* EGO03066.1 (64%) | *P. strigosozonata* EIN09898.1 (93%) | *P. strigosozonata* EIN03930.1 (59%) | P450 |
| EGO03065.1 | *S . lacrymans* EGO03790.1 (81%) | *Coniophora putanea* EIW83567.1 (53%) | *Coniophora putanea* EIW84500.1 (53%) | P450 |  | EIN09900.1 | *S . lacrymans* EGO03075.1 (76%) | *S . lacrymans* EGN97951.1 (72%) | *P. strigosozonata* EIN04702.1 (47%) | *NADBP* |
| EGO0366.1 | *P. strigosozonata* EIN09899.1 (63%) | Serpula lacrymans EGO00621.1 (38%) | *Postia placenta* XP_002469656.1 (28%) | P450 |  | EIN09901.1 | *S . lacrymans* EGO03064.1 (62%) | *P. strigosozonata* EIN06618.1 (40%) | *Postia placenda* XP_002471994.1 (40%) | GGPPS |
| EGO0367.1 | *P. strigosozonata* EIN09905.1 (71%) | *P. strigosozonata* EIN05772.1 (50%) | *Moniliophthora perniciosa XP* 002390596.1 (NA) | di-TPS |  | EIN09902.1 | *P. strigosozonata* EIN10996.1 (61%) | *P. strigosozonata* EIN14580.1 (70%) | *P. strigosozonata* EIN11653.1 (65%) | FNBP |
| EGO28833.1 | *S . lacrymans* EGO20327.1 (93%) | *S . lacrymans* EGO30581.1 (93%) | *S . lacrymans* EGO28174.1 (93%) | HP |  | EIN09903.1 | *Dichomitus squalens* EJF67049.1 (25%) | *Tramete versicolor* EIW55919.1 (27%) | *Tramete versicolor* EIW55920.1 (27%) | HP |
| EGO28834.1 | *Coprinopsis cinerea* XP_002910093.1 (36%) | *Coniophora puteana* EIW86698.1 (34%) | *Agaricus bisporus* EKV44776.1 (39%) | HP |  | EIN09904..1 | *P. strigosozonata* EIN09918.1 (33%) | *Halobacillus halophilus* YP_006180431.1 (35%) | *Gracibacillus halophilus* WP_003467270.1 (32%) | HP |
| EGN96469.1 | *S . lacrymans* EGN95321.1 (63%) | *S . lacrymans* EGO20789.1 (58%) | *S . lacrymans* EGO04694.1 (55%) | HP |  | EIN09905.1 | *S. lacrymans* EGO28832.1 (71%) | *P. strigosozonata* EIN05772.1 (55%) | *Moniliophthora perniciosa* XP 002390596.1 (NA) | di-TPS |
| EGO03072.1 | *P. strigosozonata* EIN09897.1 (73%) | *Coniosporium apollinis* EON69905.1 (33%) | *Fusarium oxysporum* EMT74057.1 (30%) | P450 |  | EIN09906.1 | *S. lacrymans* EGO03063.1 (63%) | *Rhizoctonia solani* CCO31795.1 (35%) | *Laccaria bicolor* XP_001885786.1 (31%) | P450 |
| EGO03073.1 | P*. strigosozonata* EIN09896.1 (63%) | *P. strigosozonata* EIN09907.1 (28%) | *S . lacrymans* EGO03062.1 (26%) | P450 |  | EIN09907.1 | *S. lacrymans* EGO03062.1 (66%) | *S. lacrymans* EGO03073.1 (28%) | *Ceriporiopsis subvermispora* EMD31428.1 (29%) | P450 |

^a^Homology searches were performed by using BLAST [73]. For each protein of the cluster, the percentage of identity are indicated for the three best hits.

^b^Putative functions : P450 (Cytochrome P450), GGPPS (geranylgeranyl diphosphate synthase), di-TPS (di terpene synthase), HP (hypothetical protein with no strong homology to functionally characterized proteins), NADBP (NAD(P)-binding protein), FNBP (FAD/NAD(P)-binding domain-containing protein).
