# Supplementary material for: Molecular and functional evolution of the fungal diterpene synthase genes
Source: BMC Microbiol. 2015 Oct 19;15:221. doi: 10.1186/s12866-015-0564-8 (PMC4617483; doi:10.1186/s12866-015-0564-8)
Supplement: Additional file 6: — Best BLAST hits for the genes involved in the putative B. fuckeliana and C. globosum gene cluster. (DOCX 15 kb) [file 12866_2015_564_MOESM6_ESM.docx]

**Additional file 6:**  best BLAST best hits for the genes involved in the putative *B. fuckeliana* and *C. globosum* gene cluster.

| ***B. fuckeliana*** | **Best hit^a^** | **2^nd^ Best Hit^a^** | **third Best hit^a^** | **Putative function^b^** |  | ***C. globosum*** | **Best hit^a^** | **2^nd^ Best Hit^a^** | **third Best hit^a^** | **Putative function^b^** |
| --- | --- | --- | --- | --- | --- | --- | --- | --- | --- | --- |
| *B. fuckeliana* XP_001548366.1 | *Aspergillus niger* XP_001398729.1 (68%) | *Trichoderma reesei* XP_006962948.1 (64%) | *Neosartorya fischeri* XP_001264200.1 (44%) | P450 |  | *C. globosum* XP_001227319.1 | *Thielavia terrestris* XP_003653628.1 (66%) | *Myceliophthora termophila* XP_003667270.1 (68%) | *Neurospora crassa* XP_963189.1 (59%) | P450 |
| *B. fuckeliana* XP_001548365.1 | *Aspergillus kawachii* Gaa83294.1 (34%) | *Pyrenophora tritici* XP_00190467.1 (34%) | *Aureobasidium pullulans* KEQ72097.1 (34%) | P450 |  | *C. globosum* XP_001227320.1 | ND | ND | ND | HP |
| *B. fuckeliana* XP_001548364.1 | ND | ND | ND | HP |  | *C. globosum* XP_001227321.1 | *Neosartorya fischeri* XP_001258748.1 (33%) | *Aspergillus fumigatus* EDP53544.1 (34%) | *Pseudogymnoascus destructans* ELR05338.1 (34%) | HP |
| *B. fuckeliana* XP_001548363.1 | *Pseudogymnoascus pannorum* KFY69983.1 (41%) | *Capronia epimyces* XP_007738458.1 (42%) | *Rhinocladiella mackenziei* KIX03622.1 (43%) | GST |  | *C. globosum* XP_001227322.1 | *Phaeosphaeria nodorum* XP_001801133.1 (46%) | *Aspergillus nidulans* XP_660860.1 (44% | *Eutypa lata* EMR72208.1 (60%) | P450 |
| *B. fuckeliana* XP_001548362.1 | *Endocarpon pusillum* XP_007785484.1 (56%) | *Oidiodendron maius* KIM98747.1 (46%) | *Pseudogymnoascus pannorum* KFY47812.1 (31%) | PTH11 |  | *C. globosum* XP_001227323.1 | *Aspergillus nidulans* XP_660861.1 (45%) | *Phaeosphaeria nodorum* XP_001796194.1 (46%) | *Aspergillus oryzae* XP-003190915.1 (44%) | PTH11 |
| *B. fuckeliana* XP_001548361.1 | *Fusarium oxysporum* ENH72031.1 (48%) | *Metarhiziuum anisopliae* XP_007816310.2 (46%) | *Metarhiziuum robertsii* EXU94518.1 (46%) | transposase |  | *C. globosum* XP_001227324.1 | *Aspergillus nidulans* XP_660857.1 (56% | *B. fuckeliana* CCD45418.1 (36%) | *Phaeosphaeria nodorum* XP_001801134.1 (52%) | P450 |
| *B. fuckeliana* XP_001548360.1 | ND | ND | ND | HP |  | *C. globosum* XP_001227325.1 | *Thielavia terrestris* XP_003649280.1 (77%) | *Eutypa lata* EMR70149.1 (57%) | *Aspergillus nidulans* XP_660858.1 (56%) | MSF |
| *B. fuckeliana* BC1G 13295 | *C. globosum* XP-001227327.1 (52%) | *Neurospora tetrasperma* EGO54989.1 (39%) | *Neosartorya fischeri* XP_001264196.1 (34%) | di-TPS |  | *C. globosum* XP_001227326.1 | *Podospora anserina* XP_001912715.1 (77%) | *Eutypa lata* EMR70793.1 (77%) | *Thielavia terrestris* XP_003651043.1 (76%) | GST |
|  |  |  |  |  |  | *C. globosum* XP-001227327.1 | *B. fuckeliana* BC1G 13295 (52%) | *Neurospora crassa* XP_958491.1 (43%) | *Neurospora tetrasperma* EGO54989.1 (42%) | di-TPS |

^a^Homology searches were performed by using BLAST [73]. For each protein of the cluster, the percentage of identity are indicated for the three best hits ; ND (no homology detected).

^b^Putative functions : di-TPS (di terpene synthase), P450 (Cytochrome P450), MSF (Major Facilitator Superfamily) GST (Glutathione S-transferase), PTH11 (integral membrane protein PTH11-like protein), HP (hypothetical protein with no strong homology to functionally characterized proteins).
