# Supplementary material for: Molecular and functional evolution of the fungal diterpene synthase genes
Source: BMC Microbiol. 2015 Oct 19;15:221. doi: 10.1186/s12866-015-0564-8 (PMC4617483; doi:10.1186/s12866-015-0564-8)
Supplement: Additional file 7: — Best BLAST search performed on the genes involved in the putative R. rufulum and G. lozoyensis gene cluster. (DOCX 11 kb) [file 12866_2015_564_MOESM7_ESM.docx]

**Additional file 7:** best BLAST search performed on the genes involved in the putative *R. rufulum* and *G. lozoyensis* gene cluster.

| ***R. rufulum*** | **Best hit^a^** | **2^nd^ Best Hit^a^** | **third Best hit^a^** | **Putative function^b^** |
| --- | --- | --- | --- | --- |
| NODE_2525_length_11815_cov_39.709267:525-2277 | *G. lozoyensis* EPE31713.1 (39%) | *Byssochlamys spectabilis* GAD99475.1 (25%) | *Chaetomium globosum* XP_001227362.1 (28%) | PTH11 |
| NODE_2525_length_11815_cov_39.709267:2889-4275 | *Arthroderma otae* XP_002846411.1 (49%) | *Trichophyton verrucosum* XP_003018757.1 (48%) | *Trichophyton tonsurans* EGD99954.1 (48%) | P450 |
| NODE_2525_length_11815_cov_39.709267:4666-6417 | *G. lozoyensis* EPE31715.1 (40%) | *Neosartorya fischeri* XP_001264200.1 (34%) | *Eutypa lata* EMR72744.1 (44%) | P450 |
| NODE_2525_length_11815_cov_39.709267:7075-10081 | *G. lozoyensis* EPE31716.1 (37%) | *Aspergillus niger* EHA18939.1 (34%) | *Aspergillus niger* CAK48654.1 (34%) | di-TPS |

^a^Homology searches were performed by using BLAST [73]. For each protein of the cluster, the percentage of identity are indicated for the three best hits.

^b^Putative functions : PTH11 (integral membrane protein PTH11-like protein), P450 (Cytochrome P450), di-TPS (di terpene synthase).
