# Supplementary material for: Molecular and functional evolution of the fungal diterpene synthase genes
Source: BMC Microbiol. 2015 Oct 19;15:221. doi: 10.1186/s12866-015-0564-8 (PMC4617483; doi:10.1186/s12866-015-0564-8)
Supplement: Additional file 8: — List of GGPPSs containing di-TPS Ascomycota and Basidiomycota species, early diverging lineages of fungi, and three plant species. (DOCX 23 kb) [file 12866_2015_564_MOESM8_ESM.docx]

**Additional file 8.** List of GGPPSs containing di-TPS Ascomycota and Basidiomycota species, early diverging lineages of fungi, and three plant species.

| Sequence name | Organism | Accession |
| --- | --- | --- |
|  |  |  |
| S. lacrymans_1 | *Serpula lacrymans var. lacrymans S7.3* | EGO03064.1 |
| P. strigosozonata_1 | *Punctularia strigosozonata HHB-11173 SS5* | EIN09901.1 |
| P. teres_1 | *Pyrenophora teres f. teres 0-1* | XP_003297764.1 |
| P. betae_1 | *Phoma betae* | BAD29970.1 |
| Z. tritici_1 | *Zymoseptoria tritici IPO323* | XP_003856214.1 |
| M. graminicola_1 | *Mycosphaerella graminicola* | EGP91192 |
| S. manihoticola_1 | *Sphaceloma manihoticola* | CAP07654.1 |
| A. nidulans_1 | *Aspergillus nidulans FGSC A4* | XP_659196.1 |
| A. oryzae_1 | *Aspergillus oryzae RIB40* | XP_001820658.2 |
| A. sojae_2 | *Aspergillus sojae NBRC 4239* | contig00877 |
| P. amygdali_1 | *Phomopsis amygdali* | B2DBE8.1 |
| P. amygdali_2 | *Phomopsis amygdali* | B2DBE9.1 |
| M. oryzae_1 | *Magnaporthe oryzae* | MGG_00026 |
| F. fujikuroi_1 | *Fusarium fujikuroi IMI 58289* | CCT69172.1 |
| F. proliferatum_1 | *Fusarium proliferatum* | ABC46412.2 |
| E. amarillans_1 | *Epichloe amarillans ATCC_200744* | contig_120:15299..17097 |
| E. typhina_1 | *Epichloe typhina subsp. poae E5819* | contig_78:26191..27955 |
|  |  |  |
| Asco_1 | *Saccharomyces cerevisiae S288c* | NP_015256 |
| Asco_2 | *Aspergillus clavatus NRRL 1* | XP_001268943 |
| Asco_3 | *Aspergillus clavatus NRRL 1* | XP_001275064 |
| Asco_4 | *Aspergillus clavatus NRRL 1* | XP_001276070 |
| Asco_5 | *Aspergillus clavatus NRRL 1* | XP_001275555 |
| Asco_6 | *Aspergillus flavus NRRL3357* | EED54579 |
| Asco_7 | *Aspergillus flavus NRRL3357* | EED47307 |
| Asco_8 | *Aspergillus flavus NRRL3357* | EED50215 |
| Asco_9 | *Aspergillus flavus NRRL3357* | EED53131 |
| Asco_10 | *Aspergillus flavus NRRL3357* | EED49856 |
| Asco_11 | *Aspergillus flavus NRRL3357* | EED48983 |
| Asco_12 | *Aspergillus flavus NRRL3357* | EED52171 |
| Asco_13 | *Aspergillus flavus NRRL3357* | EED51419 |
| Asco_14 | *Aspergillus fumigatus A1163* | EDP51011 |
| Asco_16 | *Aspergillus fumigatus A1163* | EDP49552 |
| Asco_17 | *Aspergillus fumigatus Af293* | XP_746966 |
| Asco_18 | *Aspergillus fumigatus Af293* | XP_752686 |
| Asco_19 | *Aspergillus fumigatus Af293* | XP_750866 |
| Asco_20 | *Aspergillus fumigatus Af293* | XP_748183 |
| Asco_21 | *Aspergillus nidulans FGSC A4* | XP_660011 |
| Asco_22 | *Aspergillus nidulans FGSC A4* | XP_660215 |
| Asco_23 | *Aspergillus nidulans FGSC A4* | XP_681412 |
| Asco_24 | *Aspergillus nidulans FGSC A4* | XP_664414 |
| Asco_25 | *Aspergillus nidulans FGSC A4* | XP_658258 |
| Asco_26 | *Aspergillus niger CBS 513.88* | XP_001391030 |
| Asco_27 | *Aspergillus niger CBS 513.88* | XP_001392584 |
| Asco_28 | *Aspergillus niger CBS 513.88* | XP_001401830 |
| Asco_29 | *Aspergillus niger CBS 513.88* | XP_001389988 |
| Asco_30 | *Aspergillus niger CBS 513.88* | XP_001397340 |
| Asco_31 | *Aspergillus niger CBS 513.88* | XP_001393187 |
| Asco_32 | *Aspergillus niger CBS 513.88* | XP_001390105 |
| Asco_33 | *Aspergillus niger CBS 513.88* | XP_001392731 |
| Asco_34 | *Glomerella graminicola M1.001* | EFQ28275 |
| Asco_35 | *Glomerella graminicola M1.001* | EFQ35158 |
| Asco_36 | *Glomerella graminicola M1.001* | EFQ25311 |
| Asco_37 | *Magnaporthe oryzae 70-15* | EHA54888 |
| Asco_38 | *Magnaporthe oryzae 70-15* | XP_364478 |
| Asco_39 | *Magnaporthe oryzae 70-15* | XP_369218 |
| Asco_40 | *Magnaporthe oryzae 70-15* | XP_001409359 |
| Asco_41 | *Magnaporthe oryzae 70-15* | XP_360889 |
| Asco_42 | *Magnaporthe oryzae 70-15* | EHA47786 |
| Asco_43 | *Magnaporthe oryzae 70-15* | XP_368486 |
| Asco_44 | *Magnaporthe oryzae 70-15* | XP_367595 |
| Asco_45 | *Magnaporthe oryzae 70-15* | EHA51611 |
| Asco_46 | *Magnaporthe oryzae 70-15* | XP_363775 |
| Asco_47 | *Metarhizium acridum CQMa 102* | EFY91456 |
| Asco_48 | *Metarhizium acridum CQMa 102* | EFY85740 |
| Asco_49 | *Metarhizium anisopliae ARSEF 23* | EFZ01791 |
| Asco_50 | *Metarhizium anisopliae ARSEF 23* | EFY96952 |
| Asco_51 | *Metarhizium anisopliae ARSEF 23* | EFZ03008 |
| Asco_52 | *Metarhizium acridum CQMa 102* | EFY87043 |
| Asco_53 | *Meyerozyma guilliermondii (Pichia guilliermondii) ATCC 6260* | EDK40664 |
| Asco_54 | *Neosartorya fischeri NRRL 181* | XP_001264444 |
| Asco_55 | *Neosartorya fischeri NRRL 181* | XP_001260721 |
| Asco_56 | *Neosartorya fischeri NRRL 181* | XP_001258098 |
| Asco_57 | *Neosartorya fischeri NRRL 181* | XP_001262975 |
| Asco_58 | *Neosartorya fischeri NRRL 181* | XP_001266369 |
| Asco_59 | *Neosartorya fischeri NRRL 181* | XP_001264204 |
| Asco_60 | *Neosartorya fischeri NRRL 181* | XP_001266466 |
| Asco_61 | *Neosartorya fischeri NRRL 181* | XP_001261884 |
| Asco_62 | *Neosartorya fischeri NRRL 181* | XP_001263940 |
| Asco_63 | *Pyrenophora teres f. teres 0-1* | XP_003298844 |
| Asco_64 | *Pyrenophora teres f. teres 0-1* | XP_003298243 |
| Asco_65 | *Talaromyces stipitatus ATCC 10500* | EED14438 |
| Asco_66 | *Talaromyces stipitatus ATCC 10500* | EED21544.1 |
| Asco_67 | *Talaromyces stipitatus ATCC 10500* | EED11493 |
| Asco_69 | *Podospora anserina S mat+* | XP_001910805.1 |
| Asco_70 | *Sclerotinia sclerotiorum 1980* | XP_001588566.1 |
| Asco_71 | *Colletotrichum higginsianum* | CCF42417.1 |
| Asco_72 | *Sporothrix schenckii ATCC 58251* | ERT02265.1 |
| Asco_73 | *Togninia minima UCRPA7* | EOO02679.1 |
| Asco_74 | *Claviceps purpurea 20.1* | CCE33183.1 |
| Asco_75 | *Yarrowia lipolytica* | XP_502923.1 |
| Asco_76 | *Gaeumannomyces graminis var. tritici R3-111a-1* | EJT80026.1 |
| Asco_77 | *Ogataea parapolymorpha DL-1* | ESX03507.1 |
| Asco_78 | *Arthrobotrys oligospora ATCC 24927* | EGX50571.1 |
| Asco_79 | *Dactylellina haptotyla CBS 200.50* | EPS45259.1 |
| Asco_80 | *Tuber melanosporum Mel28* | XP_002841728.1 |
| Asco_81 | *Pyronema omphalodes CBS 100304* | CCX06131.1 |
| Asco_82 | *Exophiala dermatitidis NIH/UT8656* | EHY61264.1 |
| Asco_83 | *Cladophialophora carrionii CBS 160.54* | ETI24027.1 |
| Asco_84 | *Cyphellophora europaea CBS 101466* | ETN43903.1 |
| Asco_85 | *Pestalotiopsis fici W106-1* | ETS81389.1 |
| Asco_86 | *Drechslerella stenobrocha 248* | EWC46615.1 |
| Asco_87 | *Botryotinia fuckeliana T4* | CCD33657.1 |
| Asco_88 | *Sordaria macrospora k-hell* | XP_003346017.1 |
| Asco_89 | *Sordaria macrospora k-hell* | CCC12492.1 |
| Asco_90 | *Glarea lozoyensis ATCC 20868* | EPE30373.1 |
| Asco_91 | *Colletotrichum higginsianum* | CCF40783.1 |
| Asco_92 | *Blumeria graminis f. sp. hordei DH14* | CCU77145 |
| Asco_93 | *Cordyceps militaris CM01* | XP_006668909.1 |
| Asco_94 | *Arthroderma gypseum CBS 118893* | XP_003173347.1 |
| Asco_95 | *Trichophyton rubrum CBS 118892* | XP_003234864.1 |
| Asco_96 | *Marssonina brunnea f. sp. 'multigermtubi' MB_m1* | EKD17112.1 |
| Asco_97 | *Nectria haematococca mpVI 77-13-4* | XP_003050856. |
| Asco_98 | *Coniosporium apollinis CBS 100218* | EON69940.1 |
| Asco_99 | *Coccidioides immitis RS* | EAS30680.2 |
| Asco_100 | *Bipolaris maydis C5* | EMD87782.1 |
|  |  |  |
| Basidio_1 | *Melampsora larici-populina 98AG31* | EGG12841 |
| Basidio_2 | *Melampsora larici-populina 98AG31* | EGG09623 |
| Basidio_3 | *Melampsora larici-populina 98AG31* | EGG12732 |
| Basidio_4 | *Melampsora larici-populina 98AG31* | EGF97904 |
| Basidio_5 | *Postia placenta Mad-698-R* | EED82802 |
| Basidio_6 | *Postia placenta Mad-698-R* | EED82616 |
| Basidio_7 | *Puccinia graminis f. sp. tritici CRL 75-36-700-3* | XP_003338205 |
| Basidio_8 | *Puccinia graminis f. sp. tritici CRL 75-36-700-3* | XP_003330784 |
| Basidio_9 | *Rhizoctonia solani AG-3 Rhs1AP* | EUC57505.1 |
| Basidio_10 | *Schizophyllum commune H4-8* | XP_003034057.1 |
| Basidio_11 | *Tremella mesenterica DSM 1558* | EIW70314.1 |
| Basidio_12 | *Fomitiporia mediterranea MF3/22* | EJD08296.1 |
| Basidio_13 | *Coniophora puteana RWD-64-598 SS2* | EIW80198.1 |
| Basidio_14 | *Fibroporia radiculosa* | CCM06424.1 |
| Basidio_15 | *Cryptococcus gattii WM276* | XP_003195557.1 |
| Basidio_16 | *Cryptococcus neoformans var. grubii H99* | AFR97264.1 |
| Basidio_17 | *Cryptococcus neoformans var. neoformans JEC21* | XP_572774.1 |
| Basidio_18 | *Piriformospora indica DSM 11827* | CCA69831.1 |
| Basidio_19 | *Mixia osmundae IAM 14324* | GAA93487.1 |
| Basidio_20 | *Sporisorium reilianum SRZ2* | CBQ69277.1 |
| Basidio_21 | *Ustilago hordei* | CCF51053.1 |
| Basidio_22 | *Ceriporiopsis subvermispora* | EMD33194.1 |
| Basidio_23 | *Moniliophthora roreri MCA 2997* | ESK88368.1 |
| Basidio_24 | *Auricularia delicata TFB-10046 SS5* | EJD49068.1 |
| Basidio_25 | *Gloeophyllum trabeum ATCC 11539* | EPQ55358.1 |
| Basidio_26 | *Laccaria bicolor S238N-H82* | XP_001889705.1 |
| Basidio_27 | *Coprinopsis cinerea okayama7#130* | XP_001829920.1 |
| Basidio_28 | *Moniliophthora roreri MCA 2997* | ESK84635.1 |
| Basidio_29 | *Rhodosporidium toruloides NP11* | EMS20556.1 |
| Basidio_30 | *Rhizoctonia solani AG-3 Rhs1AP* | EUC57505.1 |
| Basidio_31 | *Agaricus bisporus var. burnettii JB137-S8* | EKM81298.1 |
| Basidio_32 | *Fibroporia radiculosa* | CCM02947.1 |
| Basidio_33 | *Fibroporia radiculosa* | CCM03428.1 |
| Basidio_34 | *Fomitiporia mediterranea MF3/22* | EJD08296.1 |
| Basidio_35 | *Trametes versicolor FP-101664 SS1* | EIW53985.1 |
| Basidio_36 | *Coniophora puteana RWD-64-598 SS2* | EIW80501.1 |
| Basidio_37 | *Fomitopsis pinicola FP-58527 SS1* | EPS96604.1 |
| Basidio_38 | *Dichomitus squalens LYAD-421 SS1* | EJF56170.1 |
| Basidio_39 | *Stereum hirsutum FP-91666 SS1* | EIM80708.1 |
| Basidio_40 | *Fibroporia radiculosa* | CCM06424.1 |
| Basidio_41 | *Postia placenta Mad-698-R* | XP_002472170.1 |
| Basidio_42 | *Pseudozyma antarctica T-34* | GAC74473.1 |
| Basidio_43 | *Trichosporon asahii var. asahii CBS 2479* | EJT52485.1 |
| Basidio_44 | *Pseudozyma aphidis DSM 70725* | ETS62390.1 |
| Basidio_45 | *Gloeophyllum trabeum ATCC 11539* | EPQ52777.1 |
| Basidio_46 | *Dacryopinax sp. DJM-731 SS1* | EJT96922.1 |
| Basidio_47 | *Pseudozyma hubeiensis SY62* | GAC96059.1 |
| Basidio_48 | *Stereum hirsutum FP-91666 SS1* | EIM91052.1 |
| Basidio_49 | *Stereum hirsutum FP-91666 SS1* | EIM86252.1 |
| Basidio_50 | *Stereum hirsutum FP-91666 SS1* | EIM84825.1 |
| Basidio_51 | *Stereum hirsutum FP-91666 SS1* | EIM86240.1 |
| Basidio_52 | *Ustilago maydis 521* | XP_760606.1 |
| Basidio_53 | *Rhodotorula glutinis ATCC 204091* | EGU13294.1 |
| Basidio_54 | *Pseudozyma flocculosa PF-1* | EPQ26750.1 |
| Basidio_55 | *Xanthophyllomyces dendrorhous* | AAY33921 |
| Basidio_56 | *Malassezia sympodialis ATCC 42132* | CCV00380.1 |
| Basidio_57 | *Schizophyllum commune H4-8* | XP_003027369.1 |
| Basidio_58 | *Wallemia sebi CBS 633.66* | EIM23299 |
| Basidio_59 | *Moniliophthora roreri MCA 2997* | ESK95396.1 |
| Basidio_60 | *Moniliophthora roreri MCA 2997* | ESK95386.1 |
| Basidio_61 | *Moniliophthora roreri MCA 2997* | ESK96727.1 |
| Basidio_62 | *Moniliophthora perniciosa FA553* | XP_002391204.1 |
| Basidio_63 | *Serpula lacrymans var. lacrymans S7.3* | EGO00813.1 |
| Basidio_64 | *Serpula lacrymans var. lacrymans S7.3* | EGO00839.1 |
| Basidio_65 | *Punctularia strigosozonata HHB-11173 SS5* | EIN06618.1 |
|  |  |  |
| Fung_1 | *Rhizophagus irregularis DAOM 181602* | ERZ95163.1 |
| Fung_2 | *Mucor circinelloides f. circinelloides 1006PhL* | EPB92112.1 |
| Fung_3 | *Blakeslea trispora* | AFC92798.1 |
| Fung_4 | *Rhizopus delemar RA 99-880* | EIE80306.1 |
| Fung_5 | *Mucor circinelloides f. lusitanicus* | Q9P885.1 |
| Fung_6 | *Mucor circinelloides f. circinelloides 1006PhL* | EPB84486.1 |
| Fung_7 | *Batrachochytrium dendrobatidis JAM81* | XP_006682128.1 |
| Fung_8 | *Rhizopus delemar RA 99-880* | EIE75736.1 |
|  |  |  |
| Plant_1 | *Arabidopsis thaliana* | Q9ZU77.1 |
| Plant_2 | *Arabidopsis thaliana* | Q9SLG2.1 |
| Plant_3 | *Arabidopsis thaliana* | Q9LUE1.1 |
| Plant_4 | *Arabidopsis thaliana* | Q9LUD9.1 |
| Plant_5 | *Arabidopsis thaliana* | Q9LJY2.1 |
| Plant_6 | *Arabidopsis thaliana* | Q9LIA0.1 |
| Plant_7 | *Arabidopsis thaliana* | Q9LHR4.1 |
| Plant_8 | *Arabidopsis thaliana* | P34802.2 |
| Plant_9 | *Arabidopsis thaliana* | O22043.2 |
| Plant_10 | *Arabidopsis thaliana* | O04046.2 |
| Plant_11 | *Arabidopsis thaliana* | AAG40013.1 |
| Plant_12 | *Arabidopsis thaliana* | Q9LRR0.2 |
| Plant_13 | *Physcomitrella patens* | XP_001773824.1 |
| Plant_14 | *Physcomitrella patens* | XP_001752313.1 |
| Plant_15 | *Selaginella moellendorffii* | XP_002975135.1 |
| Plant_16 | *Selaginella moellendorffii* | XP_002968643.1 |
| Plant_17 | *Selaginella moellendorffii* | XP_002989893.1 |
| Plant_18 | *Zea mays* | NP_001183930.1 |
| Plant_19 | *Zea mays* | NP_001183942.1 |
| Plant_20 | *Zea mays* | NP_001183931.1 |
| Plant_21 | *Zea mays* | NP_001183944.1 |
