# Supplementary material for: Molecular and functional evolution of the fungal diterpene synthase genes
Source: BMC Microbiol. 2015 Oct 19;15:221. doi: 10.1186/s12866-015-0564-8 (PMC4617483; doi:10.1186/s12866-015-0564-8)
Supplement: Additional file 9: — Entire Phylogenetic tree generated with GGPPS. (PDF 11 kb) [file 12866_2015_564_MOESM9_ESM.pdf]

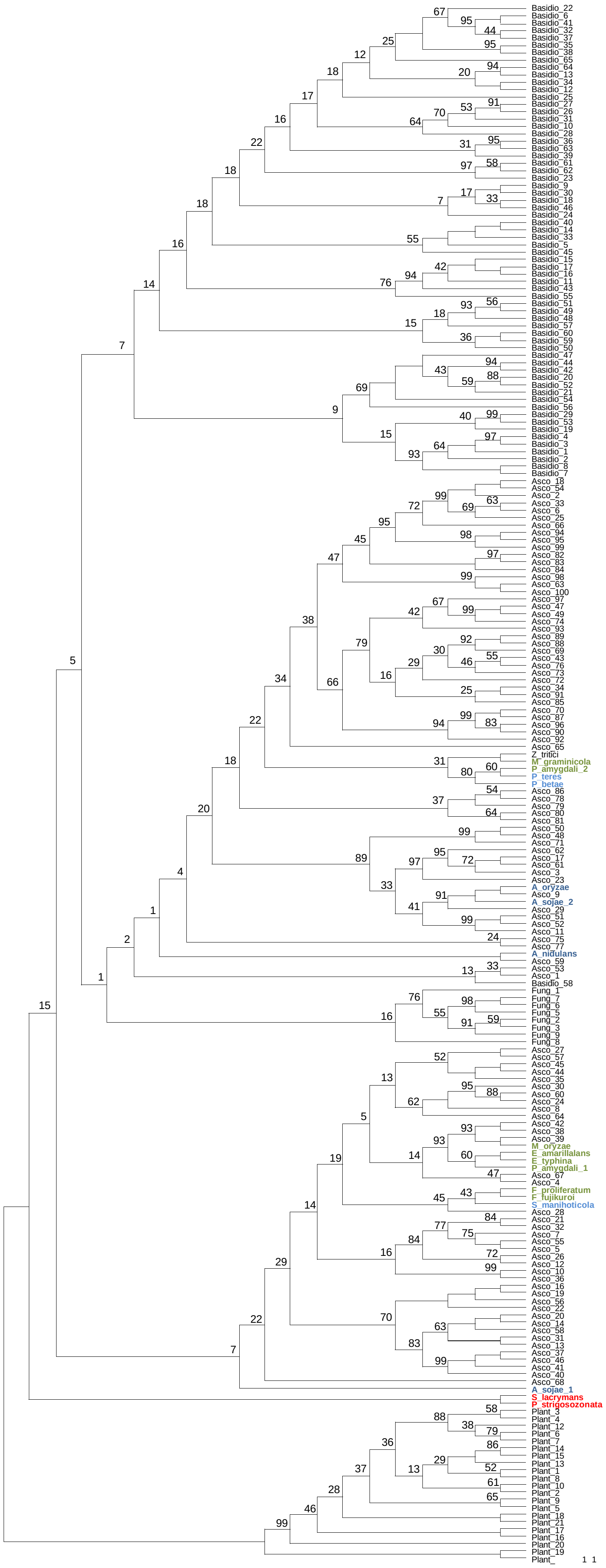

Additional file 9. Entire Phylogenetic tree generated with GGPPS. Fungal and plant GGPPS used are listed in Additional file 8. GGPPS not in di-TPS clusters are in grey colour. GGPPS in di-TPS clusters are in red for Basidiomycetes, blue for Eurotiomycetes, green for Dothideomycetes and black for Sordariomycetes. The tree was built from the alignment of 307 positions and was rooted at middle point. Bootstraps were calculated from 100 replicates. They are indicated before each node when they are not equal to 100
